# Isolated Toll-like Receptor Transmembrane Domains Are Capable of Oligomerization

**DOI:** 10.1371/journal.pone.0048875

**Published:** 2012-11-14

**Authors:** James I. Godfroy, Mohammad Roostan, Yurii S. Moroz, Ivan V. Korendovych, Hang Yin

**Affiliations:** 1 Department of Chemical and Biological Engineering, University of Colorado at Boulder, Boulder, Colorado, United States of America; 2 Department of Molecular, Cellular, and Developmental Biology, University of Colorado at Boulder, Boulder, Colorado, United States of America; 3 Department of Chemistry, Syracuse University, Syracuse, New York, United States of America; 4 Department of Chemistry and Biochemistry and BioFrontiers Institute, University of Colorado at Boulder, Boulder, Colorado, United States of America; University of Cambridge, United Kingdom

## Abstract

Toll-like receptors (TLRs) act as the first line of defense against bacterial and viral pathogens by initiating critical defense signals upon dimer activation. The contribution of the transmembrane domain in the dimerization and signaling process has heretofore been overlooked in favor of the extracellular and intracellular domains. As mounting evidence suggests that the transmembrane domain is a critical region in several protein families, we hypothesized that this was also the case for Toll-like receptors. Using a combined biochemical and biophysical approach, we investigated the ability of isolated Toll-like receptor transmembrane domains to interact independently of extracellular domain dimerization. Our results showed that the transmembrane domains had a preference for the native dimer partners in bacterial membranes for the entire receptor family. All TLR transmembrane domains showed strong homotypic interaction potential. The TLR2 transmembrane domain demonstrated strong heterotypic interactions in bacterial membranes with its known interaction partners, TLR1 and TLR6, as well as with a proposed interaction partner, TLR10, but not with TLR4, TLR5, or unrelated transmembrane receptors providing evidence for the specificity of TLR2 transmembrane domain interactions. Peptides for the transmembrane domains of TLR1, TLR2, and TLR6 were synthesized to further study this subfamily of receptors. These peptides validated the heterotypic interactions seen in bacterial membranes and demonstrated that the TLR2 transmembrane domain had moderately strong interactions with both TLR1 and TLR6. Combined, these results suggest a role for the transmembrane domain in Toll-like receptor oligomerization and as such, may be a novel target for further investigation of new therapeutic treatments of Toll-like receptor mediated diseases.

## Introduction

**Figure 1:**
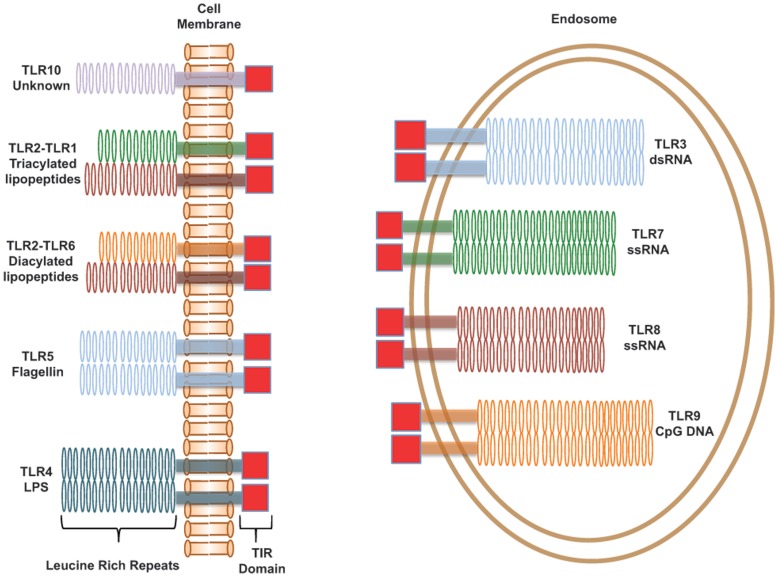
Human Toll-like Receptors. A schematic representation of the human Toll-like receptors. TLRs consist of three domains, an extracellular Leucine-rich repeat domain that recognizes the ligand, a single-pass transmembrane domain, and an intracellular TIR domain for signaling. Signaling is activated by the formation of either homodimers or heterodimers as depicted. TLRs are typically broken down into two classes, cell-surface receptors that recognize bacterial cell wall components, and endosomal receptors that recognize bacterial and viral nucleic acids.

Toll-like receptors (TLRs) are an important class of proteins involved in the innate immune response, providing the first line of defense against microbes by recognizing pathogen-associated molecular patterns (PAMPs) [1]. These receptors also play a significant role in priming adaptive immune responses [1]. TLRs are type I transmembrane proteins that consist of three domains: (1) an extracellular domain made of Leucine-rich repeats that recognizes specific PAMPs, (2) a single transmembrane domain (TMD), and (3) an intracellular Toll-interleukin 1 receptor (TIR) domain that is required for downstream signal transduction [1]. These receptors are widely conserved across species, with humans having ten known functional TLRs [2]. TLRs can be generally divided into two subgroups based on their cellular location and PAMP recognition (Fig. 1). The first subgroup is the cell surface receptors composed of TLR1, TLR2, TLR4, TLR5, TLR6, and TLR10, which recognize components of bacterial cell walls [2]. The second subgroup consists of TLR3, TLR7, TLR8, and TLR9, which are expressed in intracellular compartments like endosomes, and recognize bacterial and viral nucleic acids [2].

Over the past decade, there has been extensive work done to understand TLR structure, ligand recognition, signaling, and role in diseases. It is known that active TLRs function as either a homodimer or heterodimer, as evident by the crystal structures for TLR extracellular domains with their PAMPs for TLR3 [3], [4], TLR4 [5], TLR2/TLR1 [6], and TLR2/TLR6 [7], [8]. It is also known that the dimerization of TIR domains is required for the recruitment of various adapter proteins to initiate a signaling pathway [8]. All TLRs, except for TLR3, signal through a MyD88-dependent pathway that activates NF-κB and produces proinflammatory cytokines [8]. TLR3 signals through the TRIF pathway that produces type I interferons as well as proinflammatory cytokines [8]. Due to the outcome of their signaling pathway, the TLRs are a double-edged sword because they provide important protection from bacterial and viral pathogens, but dysregulation can lead to several disease states [9]. For example, TLR4 activation has been linked to septic shock [9], while TLR2 activation has been implicated in lupus [9], rheumatoid arthritis [10], [11], and diabetes [12], [13]. Other TLRs have also been suggested to be involved in several disease states [14], [15]. The critical importance of TLRs in various diseases has created an area of focus for new and emerging therapeutic strategies [9], [14], [16], [17].

Our interest in TLRs is to study and understand the roles of the TMDs in TLR activation. Recent research has suggested that the TMDs of proteins not only function to anchor the protein to the membrane, but that they can also play a pivotal role in membrane protein oligomerization [18]. The important role of the TMD has been demonstrated on the integrin family of proteins [19], [20], [21], [22], [23], [24], receptor tyrosine kinases [25], [26], [27], receptor-like protein tyrosine phosphatases [28], G-protein coupled receptors [29], [30], and other receptors [31], [32], [33], [34]. These reports indicate that TMD association can either be (a) the driving force for the required oligomeric state, or (b) the location of a conformational change that relates the ligand binding to signal transduction.

Recent findings have demonstrated a possible role of the TMD in TLR activation [4], [35], [36], [37], [38], [39]. First, it has been shown that by forcing the TMD and TIR domains of TLRs to be in a dimeric complex using constitutively dimeric extracellular domains, it is possible to activate the NF-κB pathway as well as other known TLR gene promoters in the absence of ligands [38], [39]. Second, FRET studies demonstrated that TLR9 existed as preformed dimers in the cell membrane and underwent small conformational changes upon ligand addition [37]. Third, structural modeling using the TLR3 extracellular domain and TLR10 TIR domain suggested a close proximity between TMD domains in the dimeric complex that could allow for the TMD region to associate [4]. Last, recent studies on TLR4 have demonstrated the importance of the tight coupling of the extracellular domain and TIR domain to the TMD as a requirement for signaling [36], and that the TLR4 extracellular domain prevents constitutive dimeric activation of TLR4 in the absence of ligand [35]. Based on these findings and the fact that the TLRs are single pass membrane proteins that are known to form functional homo- and heterodimers we asked the question if TLR TMDs are capable of oligomerization in a manner analogous to the native TLRs. Interestingly, such a hypothesis is also supported by the fact that for the two pairs of heterodimeric TLRs (TLR2/1, TLR2/6), the transmembrane domain sequences of TLR1 and TLR6 are almost identical (Table 1) due to recent evolutionary divergence [40], [41], suggesting that their TMD regions possibly contribute to their association with TLR2. Our results demonstrate that isolated TLR TMDs are indeed capable of oligomerizing and have higher propensity for TLR interactions previously identified [4], [5], [6], [7], [37], [42], [43], [44]. Studies of whether such findings apply to the full length TLRs are currently ongoing. Nonetheless, good correlations with existing structural and functional data has been observed, implying that such interactions between TLR TMDs may be biologically relevant. As such, our findings may provide new targets for the development of chemotherapeutics for diseases wherein dysregulated TLR signaling is implicated.

**Table 1:** ToxR Transmembrane Domain Sequences.

TMD	Sequence
GpA	GNRAS**LIIF** ***G*** **VMA** ***G*** **VIGTIL**GSLIN
TLR1	GNRAS**ITLLIVTIVATMLVLAVTVTSLCSYL**GSLIN
TLR2	GNRAS***A*** **LVS** ***G*** **MCC** ***A*** **LFLLILLTGVLC**GSLIN
TLR3	GNRAS**LFFMINTSILLIFIFIVLLIHF**GSLIN
TLR4	GNRAS**TIIGVSVLVVSVVAVLVYKFYF**GSLIN
TLR5	GNRAS**FSLFIVCTVTLTLFLMTILTVT**GSLIN
TLR6	GNRAS**ITLLIVTIGATMLVLAVTVTSLCIYL**GSLIN
TLR7	GNRAS**LILF** ***S*** **L** ***S*** **I** ***S*** **V** ***S*** **LFLMVMMTASHL**GSLIN
TLR8	GNRAS**VTAVILFFFTFFITTMVMLAALA**GSLIN
TLR9	GNRAS**FALSLL** ***A*** **V** ***A*** **L** ***G*** **L** ***G*** **VPMLHHL**GSLIN
TLR10	GNRAS**ALLIVTIVVIMLVLGLAVAFCCL**GSLIN
TMD5	GNRAS**WQLLAFFLAFFLDLILLIIALYL**GSLIN
Integrin α_IIb_	GNRAS**WVLV** ***G*** **VLG** ***G*** **LLLLTILVLAMW**GSLIN

Residues in regular font style are encoded by the reading frame of the plasmid constructs, capitalized bold residues are the transmembrane domain sequences studied. GpA is the glycophorin A transmembrane domain previously studied and known to have strong homotypic interactions [45], [47], [48]. TLR1-TLR10 are the transmembrane domains of the specified Toll-like receptor as identified by hydrophobic analysis. TMD5 and Integrin α_IIb_ are unrelated TMD receptors used for studying TLR specificity. Sequences of GpA, TLR2, TLR7, TLR9, and Integrin α_IIb_ all contain Small-XXX-Small motifs that have been indicated as bold italicized letters.

## Results

### TLR Transmembrane Domain Interactions in E. coli Membranes

To demonstrate proof of concept for TLR TMD interactions in both a homotypic and heterotypic manner, we used the established ToxR reporter assay [45], [46] to qualitatively assess TLR TMD interactions. The fusion proteins used in this assay consist of an extracellular maltose binding protein, which properly orients the construct to the periplasm, the TMD of interest, and a cytoplasmic cholera toxin transcriptional activation domain, ToxR. Driven by TMD-TMD interactions, dimeric ToxR domains bind the cholera toxin promoter, which induces expression of a reporter enzyme, β-galactosidase. Various plasmid constructs for this assay make it possible to monitor both homotypic [45] and heterotypic [46] interactions that are driven by TMD-TMD association (Fig. S1).

**Figure 2:**
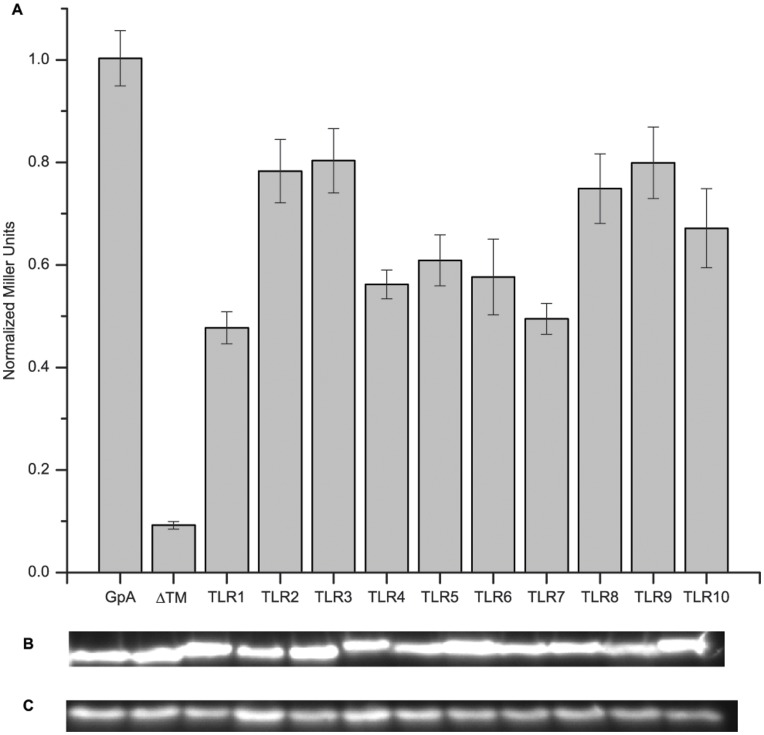
Toll-like Receptor Transmembrane Domain Homotypic Interactions. (A) The ToxR assay was used to study homotypic interactions of the TLR TMDs. A chimeric protein expressing the TMD of interest was monitored through β-galactosidase activity. In all cases, we saw that the TLRs have interaction potential solely from TMD-TMD interactions. Each TLR TMD interaction measurement analysis was performed on 3 technical replicates with >6 measurements for each replicate. Error bars depict the standard error of the mean (n – Table S1). Western blots staining for MBP were performed to monitor expression levels of the constructs – (B) chimeric maltose binding protein expression, (C) endogenous maltose binding protein expression. All samples were normalized to the GpA signal and expression levels. Significant differences were determined by use of the Tukey-Kramer test with all TLRs being significantly different than the negative control.

We first investigated homotypic interactions because the majority of the TLR family members function as homodimers. All TLR TMDs demonstrated high levels of affinity for homotypic interactions (Fig. 2A) showing 45%–85% of the activity of the positive control, glycophorin A (GpA). The TMD of GpA was used as a positive control since it has been previously demonstrated to have a very strong homotypic interaction [45], [47], [48]. A construct that does not encode a TMD and is not capable of any TMD association (ΔTM) was used as the negative control to demonstrate the background signals [49]. This ΔTM construct consists of the same maltose binding protein and ToxR domain, but lacks a TMD for proper membrane insertion (Fig. S2) [50]. Statistical analysis using the Tukey-Kramer test was performed to analyze any significant differences between all possible pairs of average interaction potentials (Table S2). All TLR TMDs were statistically different from the negative control (*p<0.0001*), indicating that the TMD-TMD interactions observed were unlikely due to systemic errors or artifacts. Among the TLRs, no discernable differences in grouping based on statistical analysis at p = 0.05 were indicated as all receptors belonged to multiple groups, except for TLR1, which with the lowest interaction potential among the family of receptors only existed in one group (Table S1).

Next, we investigated heterotypic interactions of the TLRs. We specifically studied TLR2, TLR1, and TLR6 because they are cell surface expressed receptors known to form functional heterodimers. To determine the ability of these three cell surface TLRs to have heterotypic interactions within its family, we studied the ability of the TLR1, TLR2, and TLR6 TMDs to interact with the TLR receptors also expressed at the cell surface; namely, TLR1, TLR2, TLR4, TLR5, TLR6, and TLR10. We monitored these interactions using a dominant-negative ToxR assay (Fig. S1B) which works by having a second TMD expressed with a non-functional ToxR* intracellular domain. This ToxR* domain is incapable of binding the *ctx* promoter making any interactions with this second expressed TMD unable to produce the reporter enzyme. As such, any interaction between the functional ToxR and non-functional ToxR* TMDs will result in a reduction in signal output.

**Figure 3:**
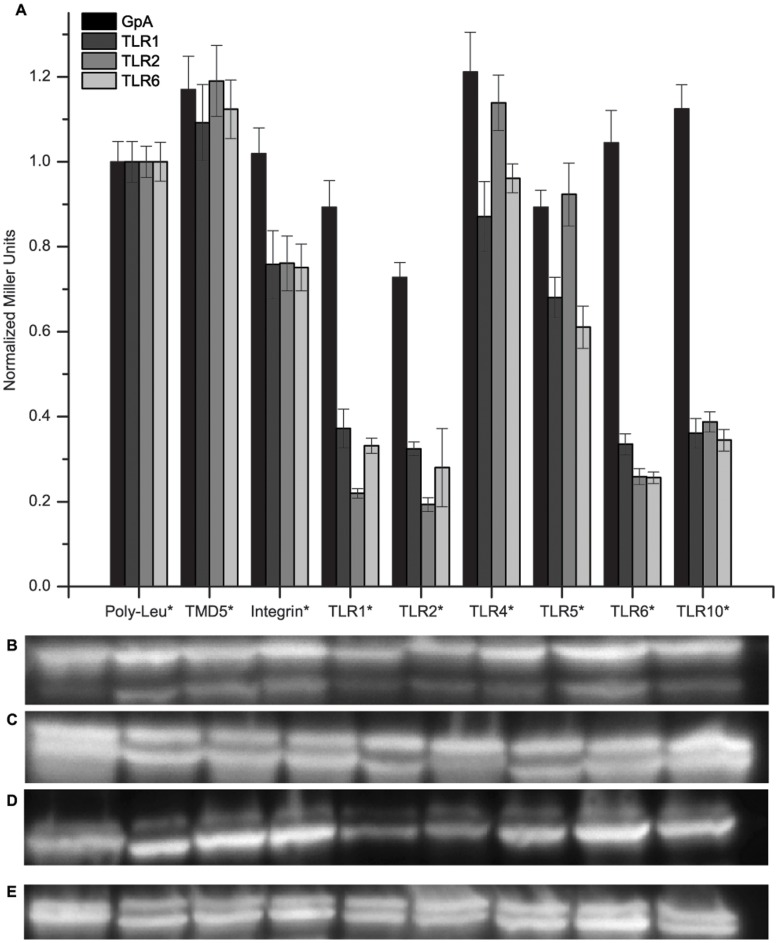
Cell- Surface Toll-like Recptor Heterotypic Interactions. (A) A dominant-negative ToxR assay was used to study heterotypic interactions of the cell-surface TLRs. Two TLR TMDs were encoded in the FHK12 *E.coli* reporter strain, one with a functional ToxR domain (dominant phenotype) and one with a nonfunctional ToxR* domain (negative phenotype). Interaction between the two different TMDs leads to a reduction in signal from that seen for homotypic interactions. The TMDs for GpA, TLR1, TLR2, and TLR6 were used with the functional ToxR domain while TMDs for poly-Leucine, TMD5, integrin α_IIb_, TLR1, TLR2, TLR4, TLR5, TLR6, and TLR10 were used with the nonfunctional ToxR* domain. Interactions were most prominent for the TLRs known to have heterotypic interactions – TLR2-TLR1 and TLR2-TLR6. TLR10 also showed strong interactions with TLR2. We also saw interaction with the same TMD further validating the homotypic interactions. Moderate interaction was seen with other TMDs that could be attributed to non-specific interactions from similar TMD motifs as completely unrelated receptors showed similar levels of knockdown. Each dominant phenotype was done in 3 technical replicates with each negative phenotype and >6 measurements made for each replicate. Error bars depict the standard error of the mean (n- Tables S3, S5, S7, S9). Western blots staining for MBP were performed to monitor expression levels of the constructs with the upper band being functional ToxR chimeras and the lower band being nonfunctional ToxR* chimeras – (B) GpA, (C) TLR1, (D) TLR2, and (E) TLR6. Significant differences for the same dominant phenotype were determined by use of the Tukey-Kramer test.

We saw a similar trend for all three TLR TMDs in the heterotypic interactions, as TLR2, TLR1 and TLR6, all showed that they had a strong interaction with TLR1, TLR2, TLR6, and TLR10, but not with TLR4 or TLR5 (Fig. 3A). Furthermore, these TMDs did not show high interaction potential with TMDs from completely unrelated receptors, TMD5 from latent membrane protein 1 and integrin α_IIb_, or with the negative control TMD of poly-Leu. Co-expression of poly-Leu as the negative phenotype for a given dominant phenotype resulted in the same signal as that seen for the dominant phenotype alone (data not shown). Therefore, poly-Leu was used as the normalization standard of 1. The strongest interactions belonged to the heterotypic interactions among TLR1, TLR2, TLR6, and TLR10, with all combinations showing >60% reduction in signal from the homotypic interaction. The same high level of knockdown was seen for the same receptor, i.e. TLR1-TLR1*, validating the homotypic signal we had seen. Interestingly these sequences, TLR1, TLR6, and TLR10, demonstrated the highest sequence similarity (Table 1) and belong to the same TLR subfamily as TLR2 [41], implying biological relevance of such an observation. Statistical analysis using the Tukey-Kramer test demonstrated TLR1, TLR2, TLR6, and TLR10 always belonged to the same grouping classification, and that classification was statistically different from all other interactions seen (Tables S5–S10). To further verify that the interaction we saw was specific, we looked at the ability of the TLR TMDs to form heterotypic interactions with unrelated transmembrane receptors and with the GpA TMD expressed as the dominant phenotype. With GpA as the dominant phenotype, we saw no significant reduction for any TMD based on statistical groupings (Table S3, S4) except TLR2, which showed a weak knockdown of 30%. The TLR1, TLR2, and TLR6 TMDs all showed a weak interaction, 30–40% knockdown, with both the integrin α_IIb_ and TLR5 TMDs, but no interaction with TMD5 or TLR4 (Tables S5–S10).

### Synthetic Transmembrane Peptide Homotypic Interactions

**Figure 4:**
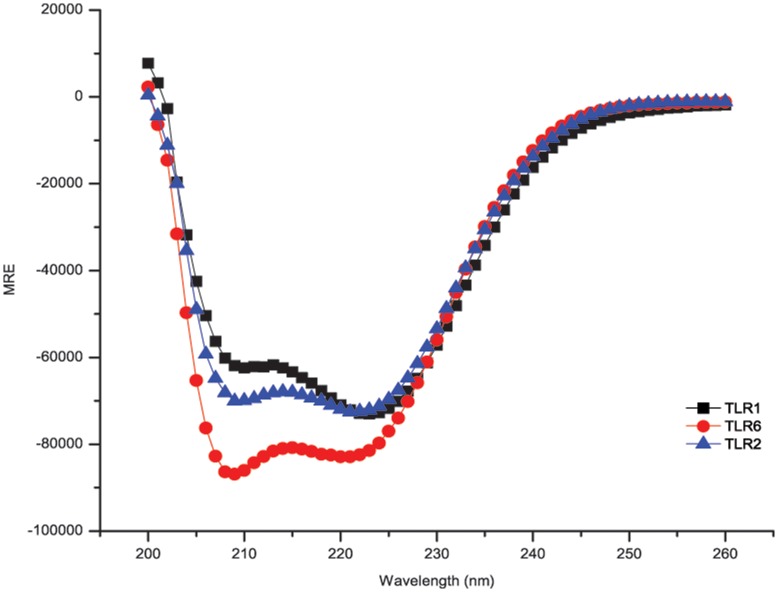
Circular Dichroism Spectra of TLR1, TLR2, and TLR6 Synthetic TMD Peptides. Far-UV spectra of the synthetic TMD peptides at concentrations ranging from 5–10 µM in the presence of 10 mM C14-betaine detergent micelles. Spectra were collected at 25°C with a step size of 1 nm and are the average of 9 scans. All peptides had helical content >99% (Table S11) as determined using CDNN [63].

To quantify the qualitative interactions demonstrated by the ToxR assay, we synthesized peptides for the TLR1, TLR2, and TLR6 TMDs. Synthetic TMD truncations have been widely used to study protein interactions in membrane mimetic systems (e.g. micelles) and may provide useful information on protein assembly in membrane bilayers [51], [52], [53]. These TLR TMD peptides were monitored in the presence of detergent micelles and found to adopt a helical conformation (Fig. 4). The helical content of the peptides was determined to be >99% for all three synthetic peptides (Table S11), indicating the micelles are a suitable mimetic system for further studies.

**Figure 5:**
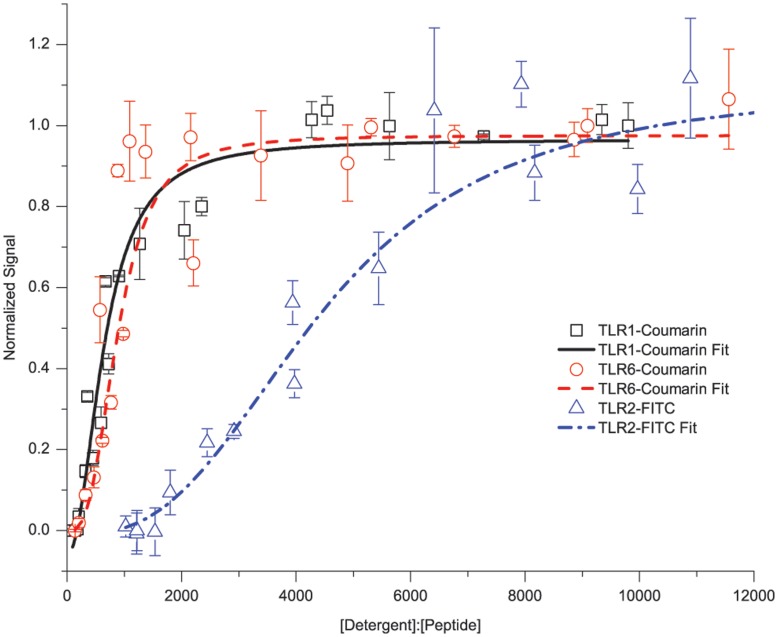
TLR1, TLR2, and TLR6 Homotypic Interactions by Fluorescence Self-Quenching. Synthetic TMD peptides were fluorescently tagged with fluorescein (TLR2) or coumarin (TLR1, TLR6). Homotypic interaction affinity was studied by changes of fluorescence intensity at different molar ratios of detergent to peptide. All samples were fixed at a peptide concentration of 1 µM, while the detergent concentration was varied. Increasing detergent competes off the TMD-TMD interactions and leads to an increase in fluorescence signal. TLR1 and TLR6 exhibit similar behavior characteristic of weak interactions as indicated by rapid release of quenched fluorescence with K_d_ of 645.63±49.08 and 883.57±86.92 respectively. TLR2 exhibits a different behavior indicative of a moderate interaction as it gradually releases quenched fluorescence with K_d_ of 4475.49±637.86. Each data point is a minimum of 3 measurements from 3 different sample preparations. Error bars are standard deviations of the measurements. K_d_ were determined using the Hill Equation.

These transmembrane peptides were fluorescently labeled with either fluorescein or 7-hydroxycoumarin, which have been previously demonstrated to exhibit fluorescence self-quenching upon interaction and do not contribute to the peptide interactions [54]. An apparent dissociation constant (K_d_) can be determined from self-quenching interactions by varying the amount of detergent present for a fixed peptide concentration. At low detergent:peptide ratios, the TMD peptides are driven to interact; however, gradually increasing the amount of detergent present at a fixed peptide concentration will cause the TMD interactions to dissociate. Then, at some critical detergent:peptide ratio, the interaction is completely dissociated and the fluorescence plateaus. For the TLR peptides we saw that TLR1 and TLR6 showed relatively weak interactions, and TLR2 showed a moderate interaction (Fig. 5). Fitting the data results in apparent dissociation constants, K_d,_ in terms of a dimensionless detergent:peptide ratio (molar fraction) as this ratio, instead of the bulk concentration, is more relevant to TMD peptide association. For TLR1 the K_d_ was determined to be 645.63±49.08, for TLR6 the K_d_ was determined to be 883.57±86.92 and for TLR2 the K_d_ was determined to be 4475.5±637.9. These molar fraction values fall within the known ranges of TMD interactions [32] and classify the TLR1 and TLR6 homotypic interactions as weak, and the TLR2 homotypic interaction as moderately strong. Further experiments were performed using sedimentation equilibrium analytical ultracentrifugation (SE-AUC) to determine oligomeric states of the TLR2 and TLR6 peptides. The TLR2 peptide was well modeled by a monomer-dimer-tetramer equilibrium (Fig. S3) and the TLR6 peptide was well modeled by a monomer-dimer equilibrium (Fig. S4) which indicated that these peptides formed oligomers in a micellar environment.

### Synthetic Transmembrane Peptide Heterotypic Interactions

**Figure 6:**
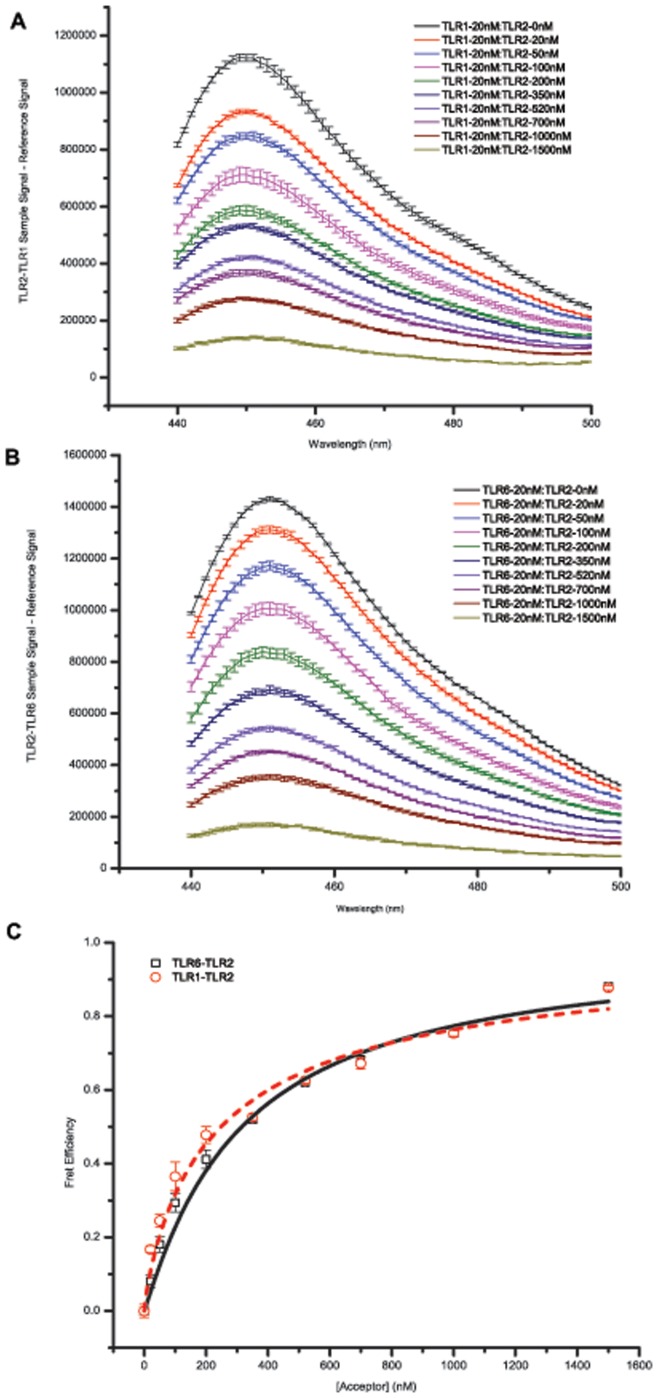
TLR2-TLR1 and TLR2-TLR6 Heterotypic Interactions Measured by Förster Resonance Energy Transfer. Synthetic TMD peptides were co-incubated in micelles with the FRET donor concentration fixed to be 20 nM and the FRET acceptor concentration varied from 0–1500 nM. The donor was excited at 415 nm and emission was monitored from 440–600 nm. Only donor emission is shown as the excitation-emission separation led to a high background signal seen in the acceptor channel. (A) TLR2-TLR1 donor channel. (B) TLR2-TLR6 donor channel. (C) FRET efficiency based on decrease in donor signal at increasing acceptor concentrations. Fitting these curves yields a TLR2-TLR1 interaction of K_d_ = 4332.0±410.7 (in molar fraction unit) and a TLR2-TLR6 interaction of K_d_ = 3490.4±190.1.

Using the same TLR1, TLR2, and TLR6 peptides, as described above, we were able to monitor the heterotypic interactions between TLR2-TLR1 and TLR2-TLR6 using a previously reported Förster resonance energy transfer (FRET) assay [22]. The titration of a fluorescein-labeled TLR2, FRET acceptor, into a 7-hydroxycoumarin labeled TLR1 or TLR6, FRET donor, resulted in the reduction of the donor emission and an appearance of the acceptor emissions, demonstrating that these transmembrane peptides have heterotypic interactions (Fig. 6). Fitting these data as described previously [25], [26], [55] it is possible to get dissociation constants. The dissociation constant for the TLR2-TLR1 interaction was 230.8±20.0 nM of acceptor labeled peptide and for TLR2-TLR6 it was 286.5±14.8 nM of acceptor labeled peptide (Fig. 6C). To make these dissociation constants comparable to the apparent dissociation constants determined by self-quenching we divided these values by the fixed detergent concentration of 1 mM to yield 4332.0±410.7 and 3490.4±190.1 for the TLR2-TLR1 and TLR2-TLR6 interactions, respectively. SE-AUC was also used to investigate the oligomeric state of the TLR2-TLR6 heterotypic interaction. Analysis showed that the molecular weight of the species decreased for TLR2 when in the presence of TLR6, suggesting that these peptides are also interacting (Fig. S5).

### TLR2 Mutational Analysis for Interface Determination

**Figure 7:**
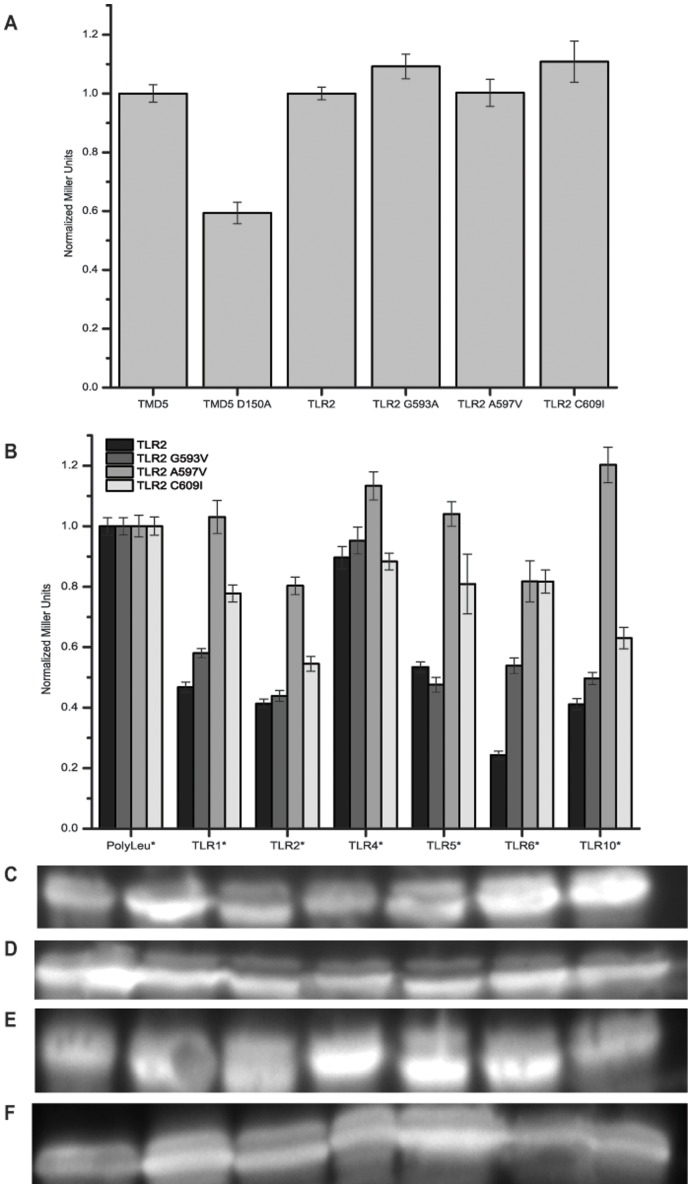
Toll-like Receptor 2 Interaction Interface Studied by Mutagenesis. TLR2 residues were mutated to study effects on homotypic and heterotypic interactions using the ToxR assay. These mutations were at positions identified in the sequence as likely interaction locations. (A) Homotypic interactions of point mutations show no difference in interaction potential from wild-type TLR2 TMD. (B) Heterotypic interactions of point mutations show the potential importance of both the Ala597 and Cys609 positions for heterotypic interactions. Western blots staining for MBP were performed to monitor expression levels of the constructs with the upper band being functional ToxR chimeras and the lower band being nonfunctional ToxR* chimeras – (C) TLR2, (D) TLR2 G593V, (E) TLR2 A597V, and (F) TLR2 C609I.

After demonstrating that the TLR2 TMD is capable of both homotypic and heterotypic interactions, we investigated residues that might be responsible for these interactions. As previous works have identified structural motifs that can be involved in TMD-TMD interactions [18], [56], we examined the TLR2 TMD sequence and found that it contained an extended Small-XXX-Small motif (Table 1, where Small can be Ala, Gly, or Ser) that has been reported to facilitate TMD dimerization [56]. Further analysis also revealed that the TMDs of TLR2, TLR1, TLR6, and TLR10 contained luminal Cys residues (Table 1). To investigate if these residues were involved in the homotypic or heterotypic interactions we performed site-directed mutagenesis at key positions in the TLR2 TMD – Gly593Val, Ala597Val, and Cys609Ile – and tested these mutants in both homotypic and heterotypic ToxR assays. The results suggest that these mutations were not critical for TLR2 homotypic interactions since no mutation demonstrated significant difference from the wild type TLR2 TMD (Fig. 7A). As a control for the effect of a point mutation on TMD interactions, we used TMD5 of latent-membrane protein 1, which has been previously studied in our laboratory and demonstrated that the Asp150 was critical for interaction [31]. For heterotypic interactions, the Ala597Val and Cys609Ile mutations played a role in heterotypic interactions since both mutations were capable of reducing the ToxR inhibition demonstrated by the native TLR2 TMD (Fig. 7B).

## Discussion

Membrane proteins account for nearly 30% of all proteins encoded in the genome and are involved in many diverse cell processes [18], [56], [57]. Previous work with TLRs has investigated the potential of the TLR TMDs and TIR domains to activate signaling pathways or gene promoters when a chimeric protein contains an extracellular domain forcing these domains into a dimeric state [38], [39]. While these data suggest that the TLRs are activated when in a dimeric state, and it has been proposed that the dimerization of the TIR domains is required for recruitment of adapter proteins in signal transduction [58], it provides no information about the role of the TMD in this dimeric interaction. Our data shows, for the first time, that isolated TLR TMDs are capable of oligomerizing both homotypically and heterotypically, showing preference for the known interaction partners of each TLR in the absence of the extracellular domain and TIR domain. These results provide critical evidence on the role of TMD interactions for the TLR family of the innate immune system and suggest that this short region of the protein likely plays an important role in the assembly and function of the receptors.

In terms of native interactions, the isolated TMDs were sufficient to recapture the known behaviors of the full-length proteins as well as provide evidence for others that are still unclassified. All TLR TMDs were capable of forming homotypic interactions (Fig. 2). These results are promising as crystal structures exist showing the extracellular domain of TLR3 [4] and of TLR4 [5] as a homodimer with their respective ligands. Additionally, a computer model based on homology modeling of the TLR3 extracellular domain and TLR10 TIR domain proposed that the TMDs could be in close proximity to interact [4]. We saw agreement with this model as the TLR3 TMD showed a very high homotypic interaction potential. It has also been shown using FRET that TLR9 exists as a preformed dimer in cell membranes and undergoes conformational changes upon binding of its ligand [37] suggesting that the TMD may be in close proximity prior to ligand activation. The ToxR assay result demonstrated that the TLR9 TMD has a very high propensity for homotypic interactions, indicating it could be a contributing factor to the pre-formed TLR9 dimers found in cells. The TLR1, TLR6, and TLR10 receptors are not known to have functional homodimers; however, TLR1 and TLR6 are known to form heterodimers with TLR2 [6], [7], [44] and while TLR10 function is still unknown, it has recently been proposed to also form a heterodimer with TLR2 [42], [43]. These known and proposed heterotypic interactions of TLR1, TLR6, and TLR10 with TLR2 were verified using our heterotypic ToxR assay (Fig. 3). The strongest heterotypic interactions were seen for the known heterotypic pairs of TLR2-TLR1 and TLR2-TLR6, as well as for the proposed TLR2-TLR10 interaction. The heterotypic interaction of TLR1, TLR2, and TLR6 with its own TMD provides further evidence for the ability of these receptors to also interact homotypically. We also saw a strong heterotypic interaction with TLR1-TLR6, TLR1-TLR10, and TLR6-TLR10 combinations, which is likely due to the high sequence similarity among these TMDs (Table 1) making these interactions analogous to the homotypic interactions. Alignments of the TLR TMD sequences showed that no TMD pairs had >25% sequence identity except for TLR1-TLR6 which has 92% sequence identity with only 2 residues different between the sequences, TLR1-TLR10 which had 50% sequence identity, and TLR6-TLR10 had 46% sequence identity. This homology is not surprising as TLR1, TLR6, and TLR10 are all on the same gene locus in humans and are the most recently diverged in phylogenetic trees [40], [41].

As a comparison, we observed weak heterotypic interactions with GpA-TLR2, TLR1-Integrin, TLR2-Integrin, TLR6-Integrin, TLR1-TLR5, and TLR6-TLR5 (Fig. 3). The GpA-TLR2 and all TLR-Integrin interactions can likely be explained by the presence of a similar Small-xxx-Small motif in the TMD sequences of GpA, TLR2, and Integrin α_IIb_ (Table 1). This motif and neighboring residues have been shown to be critical for GpA TMD interactions [48], so it is highly probable that this motif is the cause for these weak interactions. The TLR1 and TLR6 interactions with TLR5 do not appear to share any structural motifs, but as we are only looking at the isolated TMDs of these receptors, one possible explanation could be that other regions of the protein might prevent such an interaction from occurring. Our reasoning is based on the evidence that exists linking the extracellular domain as a negative regulator for interactions in other transmembrane proteins [35], [59]. One example is for a receptor-tyrosine kinase, fibroblast grown factor receptor 3 (FGFR3), whose transmembrane domain has been shown to interact independently of ligand and extracellular domains using both ToxR and biophysical assays [25], [27]. It was recently shown that the FGFR3 extracellular domain has a repulsive contribution to the overall dimerization energetics and prevents ligand-independent activation [59]. In addition to this, evidence recently demonstrated a similar trend for TLR4 [35]. It was shown that TLR4 without an extracellular domain, or with a small monomeric extracellular domain, was constitutively active. However, if the extracellular domain was bulky and monomeric, TLR4 was not constitutively active or responsive to LPS. Only with the presence of the TLR4 extracellular domain was the receptor not constitutively active and responsive to LPS [35]. These findings suggest that although some TLR TMDs demonstrate a weak heterotypic interaction potential with unexpected partners, other factors might prevent this interaction in a native context. Further studies would be needed to elucidate what are these factors contributing to the weak interactions seen.

The use of synthetic transmembrane peptides provided an alternative means to probe the interactions of TLR TMDs. We chose to focus on the TLR2, TLR1, and TLR6 TMD family as these receptors provided a method to validate both homotypic and heterotypic interactions that we saw in the ToxR assays. Additionaly, the TLR2 heterodimeric receptors have been associated with a myriad of diseases that still have unmet clinical needs [9] making this a highly interesting subfamily of TLRs. Using self-quenching and FRET studies, it was possible to determine the affinity of the various TLR2, TLR1, and TLR6 interactions. For homotypic interactions, the rapid increase in fluorescence for TLR1 and TLR6 suggested the interaction was easily driven apart while the delayed increase in TLR2 fluorescence suggested a stronger interaction (Fig. 5). The differing interaction strengths were validated by data fitting as the K_d_ were 645.63±49.08, 883.57±86.92, and 4475.5±637.9 in terms of molar fractions for TLR1, TLR6, and TLR2 respectively. These molar fraction values are in a good agreement with previously reported TMD interactions [22], [32], [48], [31]. For heterotypic interactions we saw a FRET signal for both TLR2-TLR1 and TLR2-TLR6 (Fig. 6) are also moderately strong from the respective K_d_ of 4332.0±410.7 and 3490.4±190.1. In addition to these assays, sedimentation equilibrium analytical ultracentrifugation was performed on the TLR2 and TLR6 peptides to determine oligomeric states. The TLR2 peptide showed a high molecular weight species when fit (Fig. S3) that was well modeled by a monomer-dimer-tetramer equilibrium. The TLR6 peptides also showed a high molecular weight species when fit (Fig. S4) that was well modeled by a monomer-dimer equilibrium. Also when TLR2-TLR6 were in the same sample cell, we again saw a high molecular weight species, but this species was smaller than that for TLR2 alone (Fig. S5) that suggests TLR2 and TLR6 were interacting. The results from synthetic peptide studies of TLR1, TLR2, and TLR6 validated the interactions observed in our ToxR assays and lends further support to the ability of the TLR TMDs to associate and potentially drive receptor assembly.

To further understand the TLR TMD interactions, we examined their sequences (Table 1) for any structural motifs that could provide further insight into the homotypic and heterotypic behavior as it has been demonstrated that certain structural motifs and residues are over represented in transmembrane segments and can play a pivotal role in protein-protein interactions [18], [56]. The first interesting results of this analysis was that of the strongly homotypic interactors, TLR2, TLR7, and TLR9 all contained a Small-xxx-Small motif, where small residues are considered to be Gly, Ala, or Ser. This motif occurs in over 66% of known membrane helical pairs [56] and is known to be a critical interface for the GpA dimerization and other high-affinity TMD interactions [45], [48], [49]. While this motif is commonly found in interacting transmembrane domains, it does not guarantee high interaction propensity by itself. The residues around this motif are also important in terms of stabilizing the packing interface [18]. As TLR2 showed both strong homotypic and heterotypic interactions (Figs. 2, 3, 5, 6, Figs. S3, S5), mutating residues in this interface for TLR2 would determine any role of the Small-xxx-Small motif in these interactions. Mutational analysis of potential interface residues, demonstrated that this Small-xxx-Small motif was only important for the heterotypic interactions, and was not critical for homotypic interactions (Fig. 7), suggesting that TLR2 potentially has multiple interfaces for oligomerization. Further investigations to identify and characterize the entire TLR2 TMD interface would be needed before determining the Janus interface that is critical for the previously unseen homotypic capability of TLR2.

In conclusion, we demonstrated that the TLR TMDs possess a wide range of interaction potentials and are able to recapture the known behavior of the native proteins. This work indicates a pivotal role of the TMDs in TLR dimerization, a region of this family of proteins that had previously not been studied in the same depth as the extracellular domain and TIR domain. Given the importance of TLRs in the innate immune response and its relationship to several chronic disease states, understanding the roles of TLR TMDs can provide critical insights into assembly and function of these receptor complexes and provide potential ways to regulate TLR interactions that may lead to the discovery of novel therapeutics.

## Materials and Methods

### Toll-like Receptor Transmembrane Domain Construction

As the transmembrane domain sequence is not known from crystal structures or experiment, we used hydrophobic analysis to determine the most likely consensus sequence for use in our studies. Briefly, the 10 human TLR protein sequences were accessed in FASTA format using the UniProt database (accession codes Q15399, O60603, O15455, O00206, O60602, Q9Y2C9, Q9NYK1, Q9NR97, Q9NR96, Q9BXR5) and analyzed to find the potential transmembrane domains. The programs used for predicting transmembrane domains were TMHMM, TMPred, SOSUI, DAS, and Mobyle, which were all accessed from the ExPasy topology prediction section (ca.expasay.org/tools). Residues that were identified by more than 60% of the software programs as part of the TMD were chosen as the consensus transmembrane sequences for all further studies.

### ToxR Assay Plasmid Construction

Plasmids for this assay, pTox7 and pTox6 [49], and the competent *E. coli* strain, FHK12 [61], were kindly provided by D. Langosh, Technische Universit München, Germany. The pTox7 plasmid was modified by insertion of a single base (t) after the BamH1 site to keep the proper reading frame for the designed transmembrane sequences. Oligonucleotides from Integrated DNA Technologies encoding the designed TLR1-10 TMD constructs were ligated into the Nhe1/BamH1 restriction sites of both plasmids. Mutant constructs for TLR2 were generated using site-directed mutagenesis kits (Stratagene). DNA sequencing (Genewiz, Inc., NJ) validated proper TMD insertion and reading frame.

### Homotypic ToxR Interactions

Briefly, the ToxR plasmids containing the TMD of interest (200 ng) were transformed into chemically competent FHK12 *E. coli* (200 µL) by incubating on ice for 30 min, heat shock at 42°C for 90 s, incubation on ice for 2 min, and addition of SOC media (800 µL) followed by incubation with shaking for 1 h at 37°C. This transformation mixture (50 µL) was then spread on LB agar plates containing cholaramphenicol (30 µg/mL) and ampicillin (100 µg/mL) and grown overnight at 37°C. Single colonies were selected from the plates in triplicate and added to 5 mL of LB media containing cholaramphenicol (30 µg/mL), ampicillin (100 µg/mL), and arabinose (0.0025%). These cultures were incubated overnight (16–20 h) with shaking at 37°C. The β-galactosidase activity was monitored using a Beckman Coulter DTX 880 plate-reader. First 5 µL of each culture was plated >6 times in a clear 96-well flat bottom culture plate (Sarstedt) containing 100 µL of Z-buffer/chloroform (1% β-mercaptoethanol, 10% chloroform, 89% Z buffer: 1 M sodium phosphate, 10 mM potassium chloride, 1 mM magnesium sulfate, pH 7.0). The cell densities of each well were recorded by measuring the OD_595_. Bacteria were lysed by the addition of 50 µL Z-buffer/SDS (1.6% sodium dodecyl sulfate w/v in Z-buffer) and shaking at 28°C for 10 min. Enzymatic activity was measured by adding 50 µL of Z-buffer/*ortho*-Nitrophenyl-*β*-galactosidase (ONPG) (0.4% ONPG w/v in Z-buffer) and monitoring the reaction at 405 nm for a period of 20 min at 30 s intervals. Miller units were calculated using the equation:
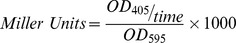



Western blotting was performed with antiserum recognizing the maltose-binding protein moiety of the constructs (Abcam). Data were normalized to GpA Miller Units and protein expression levels using ImageJ (NIH) as reported previously [48].

### Heterotypic ToxR Interactions

For heterotypic interactions one plasmid containing a functional ToxR domain (200 ng) and a second plasmid containing a non-functional ToxR* domain (200 ng) were co-transformed into chemically competent FHK12 *E. coli* (400 µL) by incubating on ice for 30 min, heat shock at 42°C for 90 s, incubation on ice for 2 min, and addition of SOC media (1600 µL) followed by incubation with shaking for 1 h at 37°C. This transformation mixture (50 µL) was then used to spread LB-agar plates containing cholaramphenicol (30 µg/mL), kanamycin (33 µg/mL), and ampicillin (100 µg/mL) and grown overnight at 37°C. Single colonies were selected from the plates and grown in 5 mL LB media containing cholaramphenicol (30 µg/mL), kanamycin (33 µg/mL), ampicillin (100 µg/mL), and arabinose (0.0025%) overnight (16–20 h) with shaking at 37°C. Analysis of activity was monitored as described in homotypic ToxR assays. Western blotting was done in the same manner as the homotypic ToxR assay with one band appearing for the functional ToxR construct and a second band for the nonfunctional ToxR* construct. Data were normalized to poly-Leu* Miller Units and corrected for varying protein expression levels using ImageJ (NIH) as previously reported [48].

### Peptide Synthesis

All peptides were synthesized at a 0.1 mmol scale on a Rink Amide resin with a loading capacity of 0.36 mmol/g using a CEM Liberty automated synthesizer with a Discovery microwave module. To increase the solubility of these highly hydrophobic peptides in polar solvents a KK sequence motif was added to both the N- and C-termini of the peptides. For all fluorophore labeling, a 6-atom flexible spacer was added to the N-terminus. The TLR2 peptide was labeled with FITC using aminohexanoic acid as the spacer using previously reported conditions [22]. The TLR1 and TLR6 peptides were labeled with coumarin using two glycines as the spacer following previously reported coupling methods [21]. For all peptides, side chain deprotection and cleavage from the resin was done using a mixture of trifluoroacetic acid/water/1,2-ethanedithiol/thioanisole/phenol/triisopropylsilane (81.5∶5:5∶2.5∶1 v/v) at room temperature under a N_2_ blanket for 2 h. The crude peptides were collected by precipitation with cold (−20°C) diethyl ether. The peptides were then purified on an Agilent 1200 series semi-preparative reverse phase high-performance liquid chromatography system with a Vydac Protein C4 column using a linear gradient of solvent A (Millipore water with 0.1% trifluoracetic acid) and solvent B (6∶3:1 isopropanol/acetonitrile/water containing 0.1% trifluoroacetic acid). The identities of the purified peptides were confirmed by MALDI-TOF mass spectrometry on a Voyager-DE-STR Biospectrometry Workstation (Perseptive Biosystems). All peptides were lyophilized using a Labconoco FreeZone 4.5 freeze drier to yield the purified peptides as their TFA salts.

### Circular Dichroism

CD measurements were performed on a ChirascanPlus spectrometer (Applied Photophysics) using a 1.0 mm path length quartz cuvette. Peptides and C14-Betaine (3-(N,N-Dimethylmyristylammonio)propanesulfonate: Sigma) were co-dissolved in 2,2,2-trifluoroethanol. The organic solvent was removed by drying to a thin film using N_2_ and further dried overnight under reduced pressure to remove all traces of TFE. Samples were resusupended in a 20 mM HEPES buffer, pH 7.4, yielding a final C14-Betaine concentration of 10 mM. Peptide concentrations were determined using Beer-Lambert law with coumarin absorbance at 400 nm using ε_400_ of 39,300 M^−1 ^cm^−1^ [62] and FITC absorbance at 495 nm using ε_495_ of 68,000 M^−1^ cm^−1^ (Invitrogen). Peptides were prepared such that final concentrations were in the 5–10 µM range. All CD spectra were measured at 25°C with a step size of 1 nm and are reported as the average of 9 scans. Data were not collected below 200 nm due to the high voltage and background noise from the C14-Betaine buffer. Helical content was determined using CDNN [63].

### Self-Quenching Assay

Fluorescence self-quenching was used to directly probe the homotypic interactions of the synthetic peptides. Fluorescently labeled peptides and C14-Betaine were co-dissolved in 2,2,2-trifluoroethanol. The organic solvent was removed using a N_2_ stream to generate a thin film of the peptide/detergent mixture and then dried over night under reduced pressure to remove all traces of organic solvent. The samples were resuspended in a 100 mM HEPES buffer, pH 7.4. Four stock solutions were prepared with final C14-Betaine concentrations of 0.15 mM, 1 mM, 5 mM, and 10 mM. Peptide concentrations of these samples were determined by UV-VIS and adjusted with the corresponding C14-Betaine only buffers such that all peptide concentrations were 1 µM. These samples were mixed in a black 96-well plate to obtain a range of peptide:detergent ratios at a fixed peptide concentration and varied detergent concentrations. Samples were allowed to equilibrate in the 96-well plate at room temperature for 2 h before measurement using a Beckman-Coulter DTX 880 Multimode Detector plate reader. The samples labeled with coumarin were excited at 360 nm and the emission filter set at 460 nm. The sample labeled with FITC was excited at 485 nm and the emission filter set at 535 nm. Each data point is blank corrected for the corresponding C14-Betaine only signal and are the average of 3 different readings. To scale the data from 0 to 1, the initial data point was averaged and set to be zero by subtracting from all further readings, and the data points that are in the plateau region were averaged and then all values were divided by this average to get a maximum of 1. The untransformed data were fit using the Hill equation using OriginPro 8.6 to get a K_d_ with the first reading being fixed as the initial signal.

### Förster Resonance Energy Transfer

FRET was used to directly probe the heterotypic association of the synthetic peptides. FRET experiments were performed on a Horiba Fluorolog-3 using a 0.3 cm path length cuvette. Peptides and C14-Betaine (1 mM) were co-dissolved in 2,2,2,-trifluoroethanol and dried under N_2_ to generate a thin film of peptide/detergent. Samples were resuspended in 100 mM HEPES, pH 7.4. Samples were titrated such that the coumarin TLR1 or TLR6 peptide concentration was fixed at 20 nM and the fluorescein TLR2 peptide concentration varied. Data were collected with an excitation wavelength of 415 nm and emission spectra were collected from 440–600 nm, with slit widths of 3 nm for both excitation and emission. Reference samples containing only fluorescein tagged TLR2 were used for calculating net FRET signals that were used in the data analysis. Data were fit using the Hill equation in OriginPro 8.6.

## Supporting Information

Figure S1 **ToxR Assay Schematic.** (A) Homotypic interactions are studied using a plasmid that encodes a TMD of interest between a functional ToxR transcriptional activator and a periplasmic directing maltose binding protein. TMD-TMD interactions leads to binding of the cholera toxin promoter that controls lacZ expression in the FHK12 *E. coli* strain used [64]. Lysis of the cells and addition of the sugar ONPG allows enzyme activity to be monitored by the production of a yellow colorimetric compound. (B) Heterotypic interactions are studied using a knockdown reporter in which two plasmids are expressed, one with the functional ToxR domain, and one with a nonfunctional ToxR* domain that contains a S87H mutation. TMD-TMD interactions involving the TMD encoded along with the ToxR* domain prevent binding of the promoter and enzyme production leading to a reduced signal. MBP – maltose binding protein, *ctx* – cholera toxin promoter, *lacZ* – β-galactosidase reporter gene.
(TIF)

Figure S2 **Control for Proper Membrane Insertion of Chimeric TLR ToxR Constructs.** Maltose deficient PD28 cells were transformed with plasmids and grown in minimal media. Growth kinetics were monitored over 3 days to determine if constructs were inserting and allowing cells to grown with maltose as the sole carbon source. Growth was observed for all TLR constructs as expected, but not for the ΔTM construct.
(TIF)

Figure S3 **TLR2 Homotypic Sedimentation Equilibrium AUC.** Sedimentation equilibrium profile at 495 nm of FITC-labeled TLR2 in density matched C14-betaine micelles (10 mM) in HEPES buffer (100 mM, pH 7.4). The partial specific volume and the solution density were fixed at 0.78730 mL/g and 1.031 g/mL, respectively. The data was analyzed using a global fitting routine. The average molecular weight obtained from the fit (14135±370 Da) corresponds well with TLR2 in a monomer-dimer-tetramer equilibrium.
(TIF)

Figure S4 **TLR6 Homotypic Sedimentation Equilibrium AUC.** Sedimentation equilibrium profile at 400 nm of coumarin-labeled TLR6 in density matched C14-betaine micelles (10 mM) in HEPES buffer (100 mM, pH 7.4). The partial specific volume and the solution density were fixed at 0.78705 mL/g and 1.031 g/mL, respectively. The data was analyzed using a global fitting routine. The average molecular weight obtained from the fit (7680±866 Da) corresponds well with TLR6 in a monomer-dimer equilibrium.
(TIF)

Figure S5 **TLR2-TLR6 Heterotypic Sedimentation Equilibrium AUC.** Sedimentation equilibrium profile at 495 nm of of FITC-labeled TLR2 in density matched C14-betaine micelles (10 mM) in HEPES buffer (100 mM, pH 7.4) in the presence of 2 equiv. of coumarin-labeled TLR6 peptide. The partial specific volume and the solution density were fixed at 0.78730 mL/g and 1.031 g/mL, respectively. The data was analyzed using a global fitting routine. The average molecular weight obtained from the fit (12330±506 Da) indicates that TLR6 shifts the monomer-dimer equilibrium of TLR2.
(TIF)

Table S1 **Homotypic TLR Interaction Grouping Information Using Tukey-Kramer Method and 95.0% Confidence Interval (p = 0.05).**
(DOC)

Table S2 **Homotypic TMD Interaction P-values Using Tukey-Kramer Method.**
(DOC)

Table S3 **GpA Heterotypic Interaction Grouping Information Using Tukey-Kramer Method and 95% Confidence Interval (p = 0.05).**
(DOC)

Table S4 **GpA TMD Heterotypic Interaction P-values Using Tukey-Kramer Method.**
(DOC)

Table S5 **TLR2 Heterotypic Interaction Grouping Information Using Tukey-Kramer Method and 95% Confidence Interval (p = 0.05).**
(DOC)

Table S6 **TLR2 Heterotypic Interaction P-values Using Tukey-Kramer Method.**
(DOC)

Table S7 **TLR1 Heterotypic Interaction Grouping Information Using Tukey-Kramer Method and 95% Confidence Interval (p = 0.05).**
(DOC)

Table S8 **TLR1 Heterotypic Interaction P-values Using Tukey-Kramer Method.**
(DOC)

Table S9 **TLR6 Heterotypic Interaction Grouping Information Using Tukey-Kramer Method and 95% Confidence Interval (p = 0.05).**
(DOC)

Table S10 **TLR6 Heterotypic Interaction P-values Using Tukey-Kramer Method.**
(DOC)

Table S11 **Helical Content Analysis of Synthetic Peptides.**
(DOC)

Text S1 **Supplementary Material and Methods.**
(DOC)
