# Supplementary figures and images for: Isolated Toll-like Receptor Transmembrane Domains Are Capable of Oligomerization
Source: PLoS One. 2012 Nov 14;7(11):e48875. doi: 10.1371/journal.pone.0048875 (PMC3498381; doi:10.1371/journal.pone.0048875)

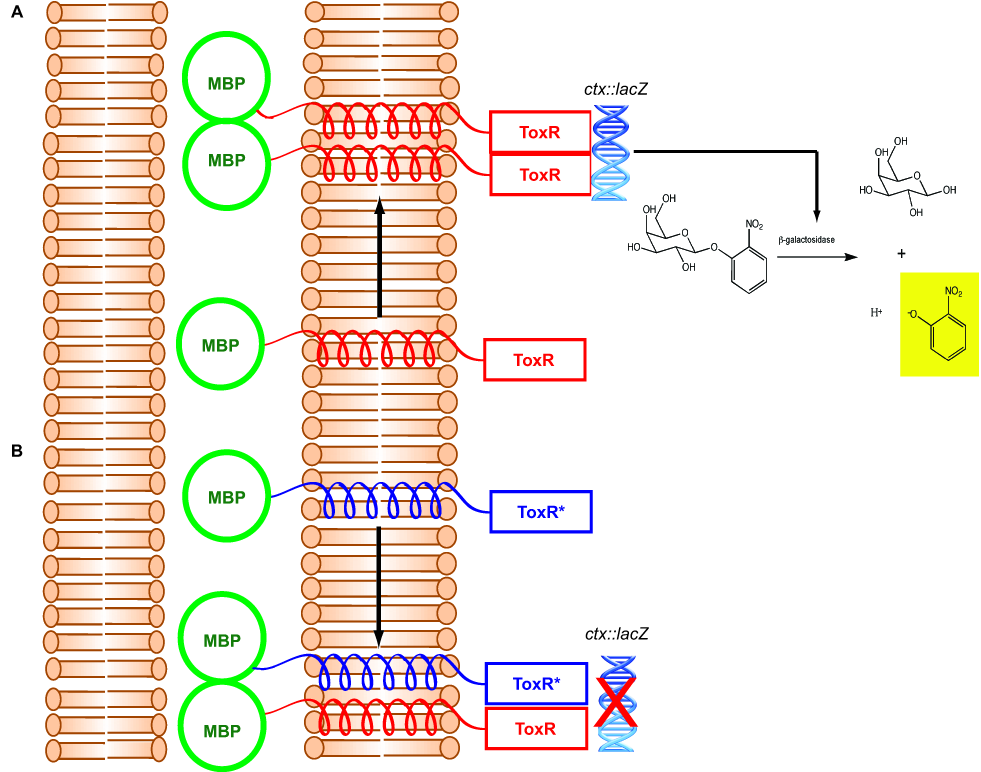

Supplement: Figure S1 — ToxR Assay Schematic. (A) Homotypic interactions are studied using a plasmid that encodes a TMD of interest between a functional ToxR transcriptional activator and a periplasmic directing maltose binding protein. TMD-TMD interactions leads to binding of the cholera toxin promoter that controls lacZ expression in the FHK12 E. coli strain used [64]. Lysis of the cells and addition of the sugar ONPG allows enzyme activity to be monitored by the production of a yellow colorimetric compound. (B) Heterotypic interactions are studied using a knockdown reporter in which two plasmids are expressed, one with the functional ToxR domain, and one with a nonfunctional ToxR* domain that contains a S87H mutation. TMD-TMD interactions involving the TMD encoded along with the ToxR* domain prevent binding of the promoter and enzyme production leading to a reduced signal. MBP – maltose binding protein, ctx – cholera toxin promoter, lacZ – β-galactosidase reporter gene. (TIF) [file pone.0048875.s001.tif]

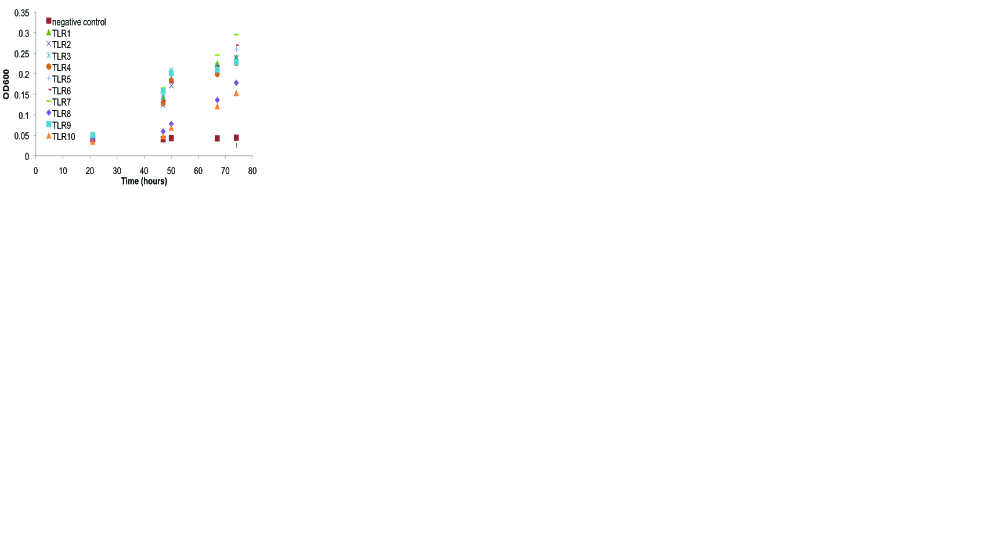

Supplement: Figure S2 — Control for Proper Membrane Insertion of Chimeric TLR ToxR Constructs. Maltose deficient PD28 cells were transformed with plasmids and grown in minimal media. Growth kinetics were monitored over 3 days to determine if constructs were inserting and allowing cells to grown with maltose as the sole carbon source. Growth was observed for all TLR constructs as expected, but not for the ΔTM construct. (TIF) [file pone.0048875.s002.tif]

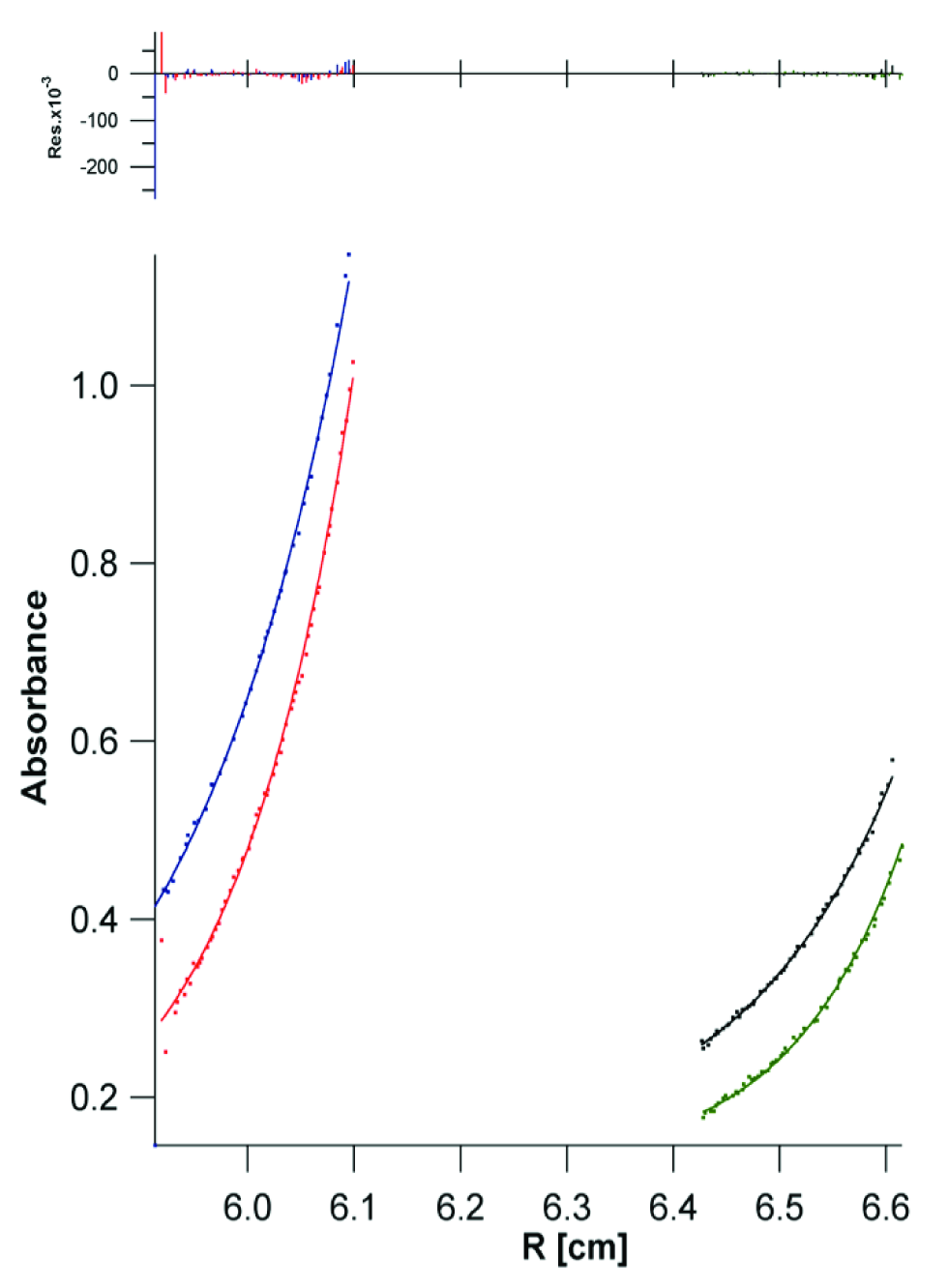

Supplement: Figure S3 — TLR2 Homotypic Sedimentation Equilibrium AUC. Sedimentation equilibrium profile at 495 nm of FITC-labeled TLR2 in density matched C14-betaine micelles (10 mM) in HEPES buffer (100 mM, pH 7.4). The partial specific volume and the solution density were fixed at 0.78730 mL/g and 1.031 g/mL, respectively. The data was analyzed using a global fitting routine. The average molecular weight obtained from the fit (14135±370 Da) corresponds well with TLR2 in a monomer-dimer-tetramer equilibrium. (TIF) [file pone.0048875.s003.tif]

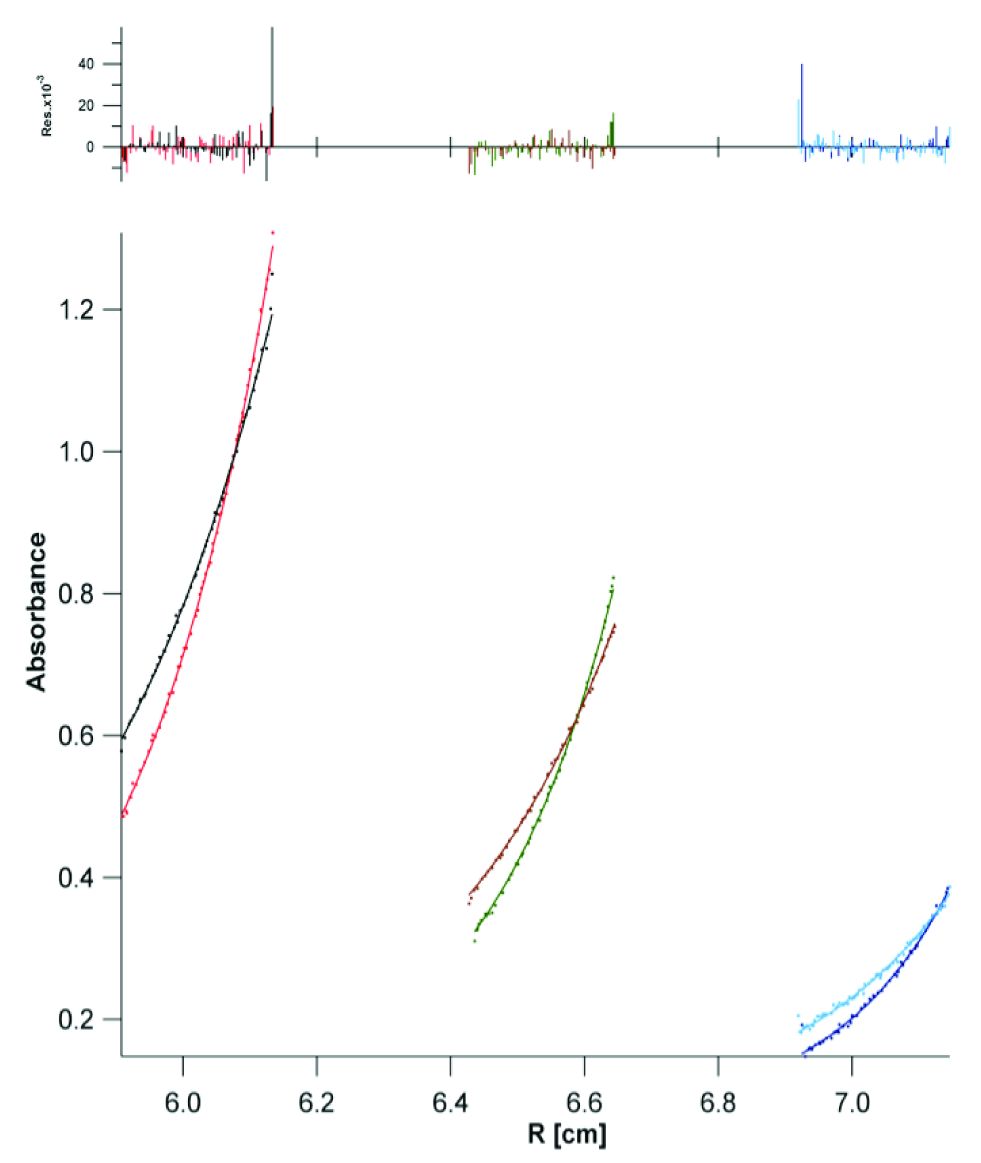

Supplement: Figure S4 — TLR6 Homotypic Sedimentation Equilibrium AUC. Sedimentation equilibrium profile at 400 nm of coumarin-labeled TLR6 in density matched C14-betaine micelles (10 mM) in HEPES buffer (100 mM, pH 7.4). The partial specific volume and the solution density were fixed at 0.78705 mL/g and 1.031 g/mL, respectively. The data was analyzed using a global fitting routine. The average molecular weight obtained from the fit (7680±866 Da) corresponds well with TLR6 in a monomer-dimer equilibrium. (TIF) [file pone.0048875.s004.tif]

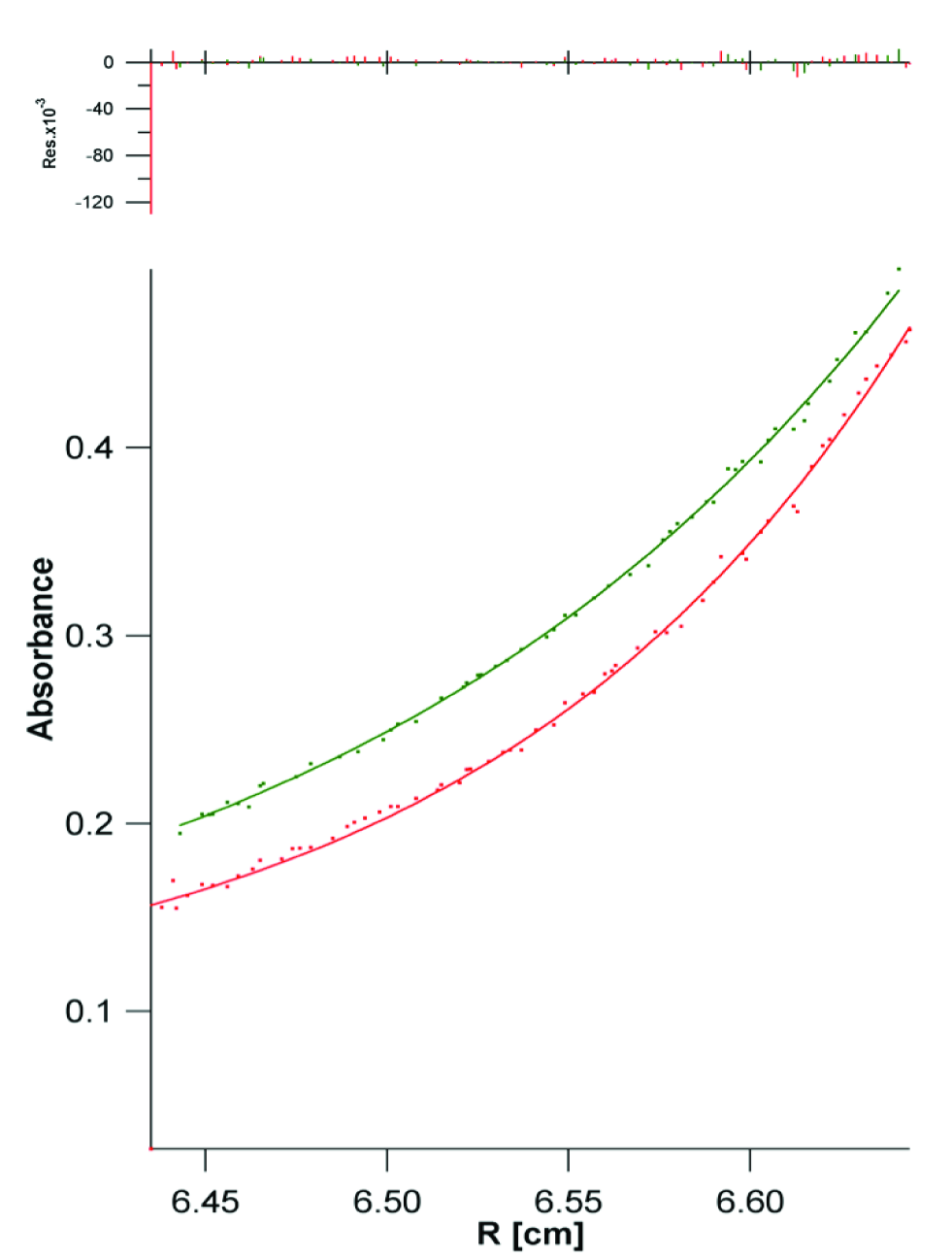

Supplement: Figure S5 — TLR2-TLR6 Heterotypic Sedimentation Equilibrium AUC. Sedimentation equilibrium profile at 495 nm of of FITC-labeled TLR2 in density matched C14-betaine micelles (10 mM) in HEPES buffer (100 mM, pH 7.4) in the presence of 2 equiv. of coumarin-labeled TLR6 peptide. The partial specific volume and the solution density were fixed at 0.78730 mL/g and 1.031 g/mL, respectively. The data was analyzed using a global fitting routine. The average molecular weight obtained from the fit (12330±506 Da) indicates that TLR6 shifts the monomer-dimer equilibrium of TLR2. (TIF) [file pone.0048875.s005.tif]
