# Supplementary material for: Isolated Toll-like Receptor Transmembrane Domains Are Capable of Oligomerization
Source: PLoS One. 2012 Nov 14;7(11):e48875. doi: 10.1371/journal.pone.0048875 (PMC3498381; doi:10.1371/journal.pone.0048875)
Supplement: Table S1 — Homotypic TLR Interaction Grouping Information Using Tukey-Kramer Method and 95.0% Confidence Interval (p = 0.05). (DOC) [file pone.0048875.s006.doc]

| **Table S1. Homotypic TLR Interaction Grouping Information Using Tukey-Kramer Method and 95.0% Confidence Interval (p = 0.05).** | | | | | | | | |
| --- | --- | --- | --- | --- | --- | --- | --- | --- |
| **TMD** | **N** | **Mean** | **Grouping^a^** | | | | | |
| *GpA* | 21 | 1.0000 | A |  |  |  |  |  |
| *ΔTM* | 22 | 0.0922 |  |  |  |  |  | F |
| *TLR1* | 22 | 0.4775 |  |  |  |  | E |  |
| *TLR2* | 17 | 0.7829 | A | B | C |  |  |  |
| *TLR3* | 22 | 0.8032 | A | B | C |  |  |  |
| *TLR4* | 22 | 0.5620 |  |  | C | D | E |  |
| *TLR5* | 23 | 0.6087 |  | B | C | D | E |  |
| *TLR6* | 21 | 0.5764 |  | B | C | D | E |  |
| *TLR7* | 22 | 0.4947 |  |  |  | D | E |  |
| *TLR8* | 19 | 0.7486 |  | B | C | D |  |  |
| *TLR9* | 23 | 0.7992 | A | B | C |  |  |  |
| *TLR10* | 19 | 0.8259 | A | B |  |  |  |  |

^a^Means that do not share a letter in grouping correspond to homotypic TMD interactions that are significantly different at 95% confidence, (p < 0.05).
