# Supplementary material for: Isolated Toll-like Receptor Transmembrane Domains Are Capable of Oligomerization
Source: PLoS One. 2012 Nov 14;7(11):e48875. doi: 10.1371/journal.pone.0048875 (PMC3498381; doi:10.1371/journal.pone.0048875)
Supplement: Table S2 — Homotypic TMD Interaction P-values Using Tukey-Kramer Method. (DOC) [file pone.0048875.s007.doc]

| **Table S2. Homotypic TMD Interaction P-values Using Tukey-Kramer Method** | | | | | | | | | | | | |
| --- | --- | --- | --- | --- | --- | --- | --- | --- | --- | --- | --- | --- |
| ***TMD*** | ***GpA*** | ***ΔTM*** | ***TLR1*** | ***TLR2*** | ***TLR3*** | ***TLR4*** | ***TLR5*** | ***TLR6*** | ***TLR7*** | ***TLR8*** | ***TLR9*** | ***TLR10*** |
| ***GpA*** | **-** | 0 | 0 | 0.1824 | 0.2156 | 0 | 0 | 0 | 0 | 0.0433 | 0.1782 | 0.4526 |
| ***ΔTM*** | 0 | **-** | 0 | 0 | 0 | 0 | 0 | 0 | 0 | 0 | 0 | 0 |
| ***TLR1*** | 0 | 0 | **-** | 0.0081 | 0.0009 | 0.9936 | 0.8362 | 0.9781 | 1.0000 | 0.0255 | 0.0009 | 0.0005 |
| ***TLR2*** | 0.1824 | 0 | 0.0081 | **-** | 1.0000 | 0.2032 | 0.5585 | 0.3131 | 0.0176 | 1.0000 | 1.0000 | 1.0000 |
| ***TLR3*** | 0.2156 | 0 | 0.0009 | 1.0000 | **-** | 0.0593 | 0.2686 | 0.1126 | 0.0024 | 0.9999 | 1.0000 | 1.0000 |
| ***TLR4*** | 0 | 0 | 0.9936 | 0.2032 | 0.0593 | **-** | 1.0000 | 1.0000 | 0.9992 | 0.4108 | 0.0626 | 0.0346 |
| ***TLR5*** | 0 | 0 | 0.8362 | 0.5585 | 0.2686 | 1.0000 | **-** | 1.0000 | 0.9309 | 0.8114 | 0.2826 | 0.1737 |
| ***TLR6*** | 0 | 0 | 0.9781 | 0.3131 | 0.1126 | 1.0000 | 1.0000 | **-** | 0.9957 | 0.5607 | 0.1190 | 0.0680 |
| ***TLR7*** | 0 | 0 | 1.0000 | 0.0176 | 0.0024 | 0.9992 | 0.9309 | 0.9957 | **-** | 0.0518 | 0.0024 | 0.0013 |
| ***TLR8*** | 0.0433 | 0 | 0.0255 | 1.0000 | 0.9999 | 0.4108 | 0.8114 | 0.5607 | 0.0518 | **-** | 1.0000 | 0.9985 |
| ***TLR9*** | 0.1782 | 0 | 0.0009 | 1.0000 | 1.0000 | 0.0626 | 0.2826 | 0.1190 | 0.0024 | 1.0000 | **-** | 1.0000 |
| ***TLR10*** | 0.4526 | 0 | 0.0005 | 1.0000 | 1.0000 | 0.0346 | 0.1737 | 0.0680 | 0.0013 | 0.9985 | 1.0000 | **-** |

Intersections correspond to the p-value for the TLR homotypic interaction being compared.
