# Supplementary material for: Isolated Toll-like Receptor Transmembrane Domains Are Capable of Oligomerization
Source: PLoS One. 2012 Nov 14;7(11):e48875. doi: 10.1371/journal.pone.0048875 (PMC3498381; doi:10.1371/journal.pone.0048875)
Supplement: Table S3 — GpA Heterotypic Interaction Grouping Information Using Tukey-Kramer Method and 95% Confidence Interval (p = 0.05). (DOC) [file pone.0048875.s008.doc]

| **Table S3. GpA Heterotypic Interaction Grouping Information Using Tukey-Kramer Method and 95% Confidence Interval (p = 0.05)** | | | | | |
| --- | --- | --- | --- | --- | --- |
| **TMD*** | **N** | **Mean** | **Grouping^a^** | | |
| *Poly-Leu** | 30 | 1.0000 | A | B |  |
| *TMD5** | 28 | 1.1703 | A |  |  |
| *Integrin** | 26 | 1.0189 | A | B |  |
| *TLR1** | 28 | 0.8934 |  | B | C |
| *TLR2** | 28 | 0.7284 |  |  | C |
| *TLR4** | 26 | 1.1905 | A |  |  |
| *TLR5** | 28 | 0.8934 |  | B | C |
| *TLR6** | 23 | 1.0451 | A | B |  |
| *TLR10** | 30 | 1.1244 | A | B |  |

^a^Means that do not share a letter in grouping correspond to heterotypic GpA-TMD* interactions that are significantly different at 95% confidence (p < 0.05).
