# Supplementary material for: Isolated Toll-like Receptor Transmembrane Domains Are Capable of Oligomerization
Source: PLoS One. 2012 Nov 14;7(11):e48875. doi: 10.1371/journal.pone.0048875 (PMC3498381; doi:10.1371/journal.pone.0048875)
Supplement: Table S4 — GpA TMD Heterotypic Interaction P-values Using Tukey-Kramer Method. (DOC) [file pone.0048875.s009.doc]

| **Table S4. GpA Heterotypic Interaction P-values Using Tukey-Kramer Method** | | | | | | | | | |
| --- | --- | --- | --- | --- | --- | --- | --- | --- | --- |
| ***TMD**** | *Poly-Leu** | *TMD5** | Integrin* | *TLR1** | *TLR2** | *TLR4** | *TLR5** | *TLR6** | *TLR10** |
| *Poly-Leu** | - | 0.5564 | 1.0000 | 0.9464 | 0.0454 | 0.4245 | 0.9464 | 0.9999 | 0.5564 |
| *TMD5** | 0.5564 | - | 0.7450 | 0.0445 | 0.0001 | 1.0000 | 0.0446 | 0.9108 | 0.9998 |
| *Integrin** | 1.0000 | 0.7450 | - | 0.8933 | 0.0337 | 0.6182 | 0.8933 | 1.0000 | 0.9546 |
| *TLR1** | 0.9464 | 0.0445 | 0.8933 | - | 0.6212 | 0.0268 | 1.0000 | 0.7763 | 0.1568 |
| *TLR2** | 0.0454 | 0.0001 | 0.0337 | 0.6212 | - | 0.0000 | 0.6211 | 0.0192 | 0.0002 |
| *TLR4** | 0.4245 | 1.0000 | 0.6182 | 0.0268 | 0.0000 | - | 0.0268 | 0.8277 | 0.9979 |
| *TLR5** | 0.9464 | 0.0446 | 0.8933 | 1.0000 | 0.6211 | 0.0268 | - | 0.7763 | 0.1569 |
| *TLR6** | 0.9999 | 0.9108 | 1.0000 | 0.7763 | 0.0192 | 0.8277 | 0.7763 | - | 0.9940 |
| *TLR10** | 0.5564 | 0.9998 | 0.9546 | 0.1568 | 0.0002 | 0.9979 | 0.1569 | 0.9940 | - |

Intersections correspond to the p-value for the GpA-TMD* heterotypic interaction being compared.
