# Supplementary material for: Isolated Toll-like Receptor Transmembrane Domains Are Capable of Oligomerization
Source: PLoS One. 2012 Nov 14;7(11):e48875. doi: 10.1371/journal.pone.0048875 (PMC3498381; doi:10.1371/journal.pone.0048875)
Supplement: Table S5 — TLR2 Heterotypic Interaction Grouping Information Using Tukey-Kramer Method and 95% Confidence Interval (p = 0.05). (DOC) [file pone.0048875.s010.doc]

| **Table S5. TLR2 Heterotypic Interaction Grouping Information Using Tukey-Kramer Method and 95% Confidence Interval (p = 0.05)** | | | | | | |
| --- | --- | --- | --- | --- | --- | --- |
| **TMD*** | **N** | **Mean** | **Grouping^a^** | | | |
| *Poly-Leu** | 30 | 1.000 | A | B |  |  |
| *TMD5** | 21 | 1.1904 | A |  |  |  |
| *Integrin** | 19 | 0.7609 |  |  | C |  |
| *TLR1** | 26 | 0.2198 |  |  |  | D |
| *TLR2** | 25 | 0.1930 |  |  |  | D |
| *TLR4** | 23 | 1.1386 | A |  |  |  |
| *TLR5** | 25 | 0.9233 |  | B | C |  |
| *TLR6** | 30 | 0.2588 |  |  |  | D |
| *TLR10** | 26 | 0.3877 |  |  |  | D |

^a^Means that do not share a letter in grouping correspond to TLR2-TMD* interactions that are significantly different at 95% confidence (p < 0.05).
