# Supplementary material for: Isolated Toll-like Receptor Transmembrane Domains Are Capable of Oligomerization
Source: PLoS One. 2012 Nov 14;7(11):e48875. doi: 10.1371/journal.pone.0048875 (PMC3498381; doi:10.1371/journal.pone.0048875)
Supplement: Table S6 — TLR2 Heterotypic Interaction P-values Using Tukey-Kramer Method. (DOC) [file pone.0048875.s011.doc]

| **Table S6. TLR2 Heterotypic Interaction P-values Using Tukey-Kramer Method** | | | | | | | | | |
| --- | --- | --- | --- | --- | --- | --- | --- | --- | --- |
| ***TMD**** | *Poly-Leu** | *TMD5** | Integrin* | *TLR1** | *TLR2** | *TLR4** | *TLR5** | *TLR6** | *TLR10** |
| *Poly-Leu** | - | 0.1080 | 0.0179 | 0.0000 | 0.0000 | 0.4574 | 0.9546 | 0.0000 | 0.0000 |
| *TMD5** | 0.1080 | - | 0.0000 | 0.0000 | 0.0000 | 0.9983 | 0.0050 | 0.0000 | 0.0000 |
| *Integrin** | 0.0179 | 0.0000 | - | 0.0000 | 0.0000 | 0.0000 | 0.3651 | 0.0000 | 0.0000 |
| *TLR1** | 0.0000 | 0.0000 | 0.0000 | - | 1.0000 | 0.0000 | 0.0000 | 0.9995 | 1.0000 |
| *TLR2** | 0.0000 | 0.0000 | 0.0000 | 1.0000 | - | 0.0000 | 0.0000 | 0.9822 | 0.0816 |
| *TLR4** | 0.4574 | 0.9983 | 0.0000 | 0.0000 | 0.0000 | - | 0.0449 | 0.0000 | 0.0000 |
| *TLR5** | 0.9546 | 0.0050 | 0.3651 | 0.0000 | 0.0000 | 0.0449 | - | 0.0000 | 0.0000 |
| *TLR6** | 0.0000 | 0.0000 | 0.0000 | 0.9995 | 0.9822 | 0.0000 | 0.0000 | - | 0.5136 |
| *TLR10** | 0.0000 | 0.0000 | 0.0000 | 0.2033 | 0.0816 | 0.0000 | 0.0000 | 0.5136 | - |

Intersections correspond to the p-value for the TLR2-TMD* heterotypic interaction being compared.
