# Supplementary material for: Isolated Toll-like Receptor Transmembrane Domains Are Capable of Oligomerization
Source: PLoS One. 2012 Nov 14;7(11):e48875. doi: 10.1371/journal.pone.0048875 (PMC3498381; doi:10.1371/journal.pone.0048875)
Supplement: Table S8 — TLR1 Heterotypic Interaction P-values Using Tukey-Kramer Method. (DOC) [file pone.0048875.s013.doc]

| **Table S8. TLR1 Heterotypic Interaction P-values Using Tukey-Kramer Method** | | | | | | | | | |
| --- | --- | --- | --- | --- | --- | --- | --- | --- | --- |
| ***TMD**** | *Poly-Leu** | *TMD5** | Integrin* | *TLR1** | *TLR2** | *TLR4** | *TLR5** | *TLR6** | *TLR10** |
| *Poly-Leu** | - | 0.9673 | 0.0665 | 0.0000 | 0.0000 | 0.8026 | 0.0018 | 0.0000 | 0.0000 |
| *TMD5** | 0.9673 | - | 0.0013 | 0.0000 | 0.0000 | 0.1386 | 0.0000 |  | 0.0000 |
| *Integrin** | 0.0665 | 0.0013 | - | 0.0001 | 0.0000 | 0.9031 | 0.9881 | 0.0000 | 0.0000 |
| *TLR1** | 0.0000 | 0.0000 | 0.0001 | - | 0.9996 | 0.0000 | 0.0036 | 0.9999 | 1.0000 |
| *TLR2** | 0.0000 | 0.0000 | 0.0000 | 0.9996 | - | 0.0000 | 0.0002 | 1.0000 | 1.0000 |
| *TLR4** | 0.8026 | 0.1386 | 0.9031 | 0.0000 | 0.0000 | - | 0.2914 | 0.0000 | 0.0000 |
| *TLR5** | 0.0018 | 0.0000 | 0.9881 | 0.0036 | 0.0002 | 0.2914 | - | 0.0000 | 0.0020 |
| *TLR6** | 0.0000 | 0.0000 | 0.0000 | 0.9999 | 1.0000 | 0.0000 | 0.0004 | - | 1.0000 |
| *TLR10** | 0.0000 | 0.0000 | 0.0000 | 1.0000 | 1.0000 | 0.0000 | 0.0020 | 1.0000 | - |

Intersections correspond to the p-value for the TLR1-TMD* heterotypic interaction being compared.
