# Supplementary material for: Isolated Toll-like Receptor Transmembrane Domains Are Capable of Oligomerization
Source: PLoS One. 2012 Nov 14;7(11):e48875. doi: 10.1371/journal.pone.0048875 (PMC3498381; doi:10.1371/journal.pone.0048875)
Supplement: Table S10 — TLR6 Heterotypic Interaction P-values Using Tukey-Kramer Method. (DOC) [file pone.0048875.s015.doc]

| **Table S10. TLR6 Heterotypic Interaction P-values Using Tukey-Kramer Method** | | | | | | | | | |
| --- | --- | --- | --- | --- | --- | --- | --- | --- | --- |
| ***TMD**** | *Poly-Leu** | *TMD5** | Integrin* | *TLR1** | *TLR2** | *TLR4** | *TLR5** | *TLR6** | *TLR10** |
| *Poly-Leu** | - | 0.0000 | 0.4378 | 0.0000 | 0.0000 | 0.9990 | 0.0000 | 0.0000 | 0.0000 |
| *TMD5** | 0.4378 | - | 0.0000 | 0.0000 | 0.0000 | 0.0930 | 0.0000 | 0.8671 | 0.0000 |
| *Integrin** | 0.0007 | 0.0000 | - | 0.0000 | 0.0000 | 0.0079 | 0.2792 | 0.0000 | 0.0000 |
| *TLR1** | 0.0000 | 0.0000 | 0.0000 | - | 0.9932 | 0.0000 | 0.0001 | 0.9287 | 1.0000 |
| *TLR2** | 0.0000 | 0.0000 | 0.0000 | 0.9932 | - | 0.0000 | 0.0000 | 1.0000 | 0.9712 |
| *TLR4** | 0.9990 | 0.0930 | 0.0079 | 0.0000 | 0.0000 | - | 0.0000 | 0.0000 | 0.0000 |
| *TLR5** | 0.0000 | 0.0000 | 0.2792 | 0.0001 | 0.0000 | 0.0000 | - | 0.0000 | 0.0002 |
| *TLR6** | 0.0000 | 0.8671 | 0.0000 | 0.9287 | 1.0000 | 0.0000 | 0.0000 | - | 0.8390 |
| *TLR10** | 0.0000 | 0.0000 | 0.0000 | 1.0000 | 0.9712 | 0.0000 | 0.0002 | 0.8390 | - |

Intersections correspond to the p-value for the TLR6-TMD* heterotypic interaction being compared.
