# Supplementary material for: Isolated Toll-like Receptor Transmembrane Domains Are Capable of Oligomerization
Source: PLoS One. 2012 Nov 14;7(11):e48875. doi: 10.1371/journal.pone.0048875 (PMC3498381; doi:10.1371/journal.pone.0048875)
Supplement: Table S11 — Helical Content Analysis of Synthetic Peptides. (DOC) [file pone.0048875.s016.doc]

| **Table S11. Helical Content Analysis of Synthetic Peptides.** | | | |
| --- | --- | --- | --- |
| **TLR1** | ***200-260 nm*** | ***205-260 nm*** | ***210-260 nm*** |
| *Helix* | 99.70% | 99.90% | 99.90% |
| *Antiparallel* | 0.00% | 0.00% | 0.00% |
| *Parallel* | 0.10% | 0.00% | 0.10% |
| *Beta-Turn* | 2.60% | 2.40% | 2.20% |
| *Rndm. Coil* | 0.40% | 0.20% | 0.30% |
| *Total Sum* | 102.80% | 102.60% | 102.50% |
| **TLR2** | ***200-260 nm*** | ***205-260 nm*** | ***210-260 nm*** |
| *Helix* | 99.80% | 99.90% | 99.90% |
| *Antiparallel* | 0.00% | 0.00% | 0.00% |
| *Parallel* | 0.00% | 0.00% | 0.10% |
| *Beta-Turn* | 2.50% | 2.20% | 2.00% |
| *Rndm. Coil* | 0.30% | 0.20% | 0.30% |
| *Total Sum* | 102.60% | 102.40% | 102.30% |
| **TLR6** | ***200-260 nm*** | ***205-260 nm*** | ***210-260 nm*** |
| *Helix* | 99.90% | 100.00% | 100.00% |
| *Antiparallel* | 0.00% | 0.00% | 0.00% |
| *Parallel* | 0.00% | 0.00% | 0.00% |
| *Beta-Turn* | 2.00% | 1.70% | 1.60% |
| *Rndm. Coil* | 0.10% | 0.10% | 0.20% |
| *Total Sum* | 102.10% | 101.80% | 101.80% |
