# Supplementary material for: Isolated Toll-like Receptor Transmembrane Domains Are Capable of Oligomerization
Source: PLoS One. 2012 Nov 14;7(11):e48875. doi: 10.1371/journal.pone.0048875 (PMC3498381; doi:10.1371/journal.pone.0048875)
Supplement: Text S1 — Supplementary Material and Methods. (DOC) [file pone.0048875.s017.doc]

**Supplementary Material and Methods**

***Proper Membrane Insertion of Chimeric Proteins***

To verify that the TMD constructs for the Toll-like receptors were being properly integrated in the bacterial membrane, a minimal media growth assay was performed. In this assay, a bacterial strain that lacks the maltose protein, PD28 [1], were transformed with the plasmids encoding the TMD of interest. The PD28 bacteria were grown in minimal media with maltose as the sole carbon source. Cell density was monitored at OD_595_ and corresponds to the efficiency of membrane integration. We saw that all designed TLR TMDs were able to properly insert into the bacterial membranes as indicated by the increasing OD_595_ (Figure S2). The ΔTM construct was not capable of inserting and showed no growth as was expected.

***Analytical Ultracentrifugation***

Equilibrium sedimentation was used primarily to determine the association state of TLR2, TLR6, and TLR2-TLR6. The experiments were performed in a Beckman XL-I analytical ultracentrifuge (Beckman Coulter) using six-channel carbon-epoxy composite centerpieces at 25 °C. Peptides were co-dissolved in trifluoroethanol (TFE) and C14-betaine. The organic solvent was removed using dry nitrogen gas and the resulting thin film of peptide/detergent mixture was dried overnight. The sample was then dissolved in buffer previously determined to match the density of the detergent component (100 mM HEPES buffer (pH= 7.4) buffer containing 25% D2O). The final concentration of C14-betaine is 10 mM in all of the samples. Peptide concentrations were determined by UV-Vis on the Beckman Coulter XL-I and dilutions were carried out to get concentrations in the desired absorbance ranges of the instrument (0.3-1) using the following extinction coefficients: 39,300 M^-1^cm^-1^ at 400 nm for coumarin labeled peptides and 69,000 M^-1^cm^-1^ at 495 nm for FITC labeled peptides. Data at different measurement speeds (30, 35 krpm) and different peptide:detergent ratios were analyzed by global curve-fitting of radial concentration gradients (measured using optical absorption) to the sedimentation equilibrium equation. Absorbance at both 400 nm and 495 nm was measured. Peptide partial specific volumes were calculated using previously described methods [2] and residue molecular weights corrected for the 25% D2O exchange expected for the density-matched buffer. The solvent density (1.031 g/ml) was calculated using the program Sednterp [3]. These coefficients were multiplied by the molar detergant ratio concentration units.

In order to estimate the association state of the peptides, the sedimentation equilibrium was fitted to a single species model:

where *E* = baseline (zero concentration) absorbance, *c_o_* is the peptide/detergent ratio of the peptide at *r_o_*, *ε* is the molar extinction coefficient, *l* is the optical path length, ω =2π*RPM, *R*= 8.3144 ×10^7^ erg K^-1^mol^-1^ , *T* is temperature in K, *M* is the buoyant molecular weight of the peptide.

Molecular weight was obtained from the buoyant molecular weight using:

where *M* is the buoyant molecular weight, $\overset{-}{\nu}$ is the partial specific volume, and $\rho$ is the solution density*.*

The results showed that TLR2 exists in a monomer-dimer-tetramer equilibrium and TLR6 exists in a monomer-dimer equilibrium. For the heterotypic interaction, when TLR2 and TLR6 were both present in the same sample cell, the apparent smaller average molecular weight of the species monitored at 495 nm (where only TLR2 absorbs) indicates that TLR2 and TLR6 are also interacting. The resulting fits are shown in Figs. S3-S5.

1. Duplay P, Szmelcman S, Bedouelle H, Hofnung M (1987) Silent and functional changes in the periplasmic matlose-binding protein of Escherichia coli K12. I. Transport of maltose. Journal of Molecular Biology 194: 663-673.

2. Durchschlag H, Zipper P (1994) Calculation of the partial volume of organic compounds and polymers. Progress in Colloid and Polymer Science 94: 20-39.

3. Lebowitz J, Lewis MS, Schuck P (2002) Modern analytical ultracentrifugation in protein science: A tutorial review. Protein Science 11: 2067-2079.
